# Fufang Huangbo Formula mitigates myeloproliferative neoplasms by activating p53/p21 signaling axis and inhibiting STAT3 and NF-κB signaling pathways

**DOI:** 10.1016/j.pscia.2026.100121

**Published:** 2026-04-16

**Authors:** Mingjie Liu, Yanxia Li, Chengxue Qin, Lingling Wang, Meiqi Guo, Yi Wang, Qian Zhou, Zhida Shi, Weimin Hao, Yuan Li, Baobing Zhao

**Affiliations:** a State Key Laboratory of Discovery and Utilization of Functional Components in Traditional Chinese Medicine, Shandong University, Jinan, Shandong, 250012, China; b Key Lab of Chemical Biology (MOE), Shandong University, Jinan, Shandong, 250012, China; c NMPA Key Laboratory for Technology Research and Evaluation of Drug Products, Shandong University, Jinan, Shandong, 250012, China; d Department of Pharmacology, School of Pharmaceutical Sciences, Cheeloo College of Medicine, Shandong University, Jinan, Shandong, 250012, China; e Research Center for Traditional Chinese Medicine and Clinical Pharmacy, Shandong Provincial Maternal and Child Health Care Hospital, Jinan, Shandong, 250012, China; f Department of Spine Surgery, Heze Municipal Hospital, 2888 Caozhou Road, Heze, Shandong, 274031, China

**Keywords:** Myeloproliferative neoplasms, Fufang Huangbo Formula, Senescence, p53/p21, NF-κB, STAT3

## Abstract

Myeloproliferative neoplasms (MPN) are hematological disorders driven by mutated hematopoietic stem cells, characterized by an increased risk of thrombosis and progression to leukemia. Patients with MPN exhibit elevated levels of inflammatory factors, which function as promoters of disease progression and transformation into acute leukemia. Fufang Huangbo formula (FHF) is a classic traditional Chinese herbal medicine widely used in the treatment of inflammation-related diseases, where it has shown considerable therapeutic efficacy. However, its potential effects on MPNs remain unclear. In this study, we demonstrate for the first time across multiple animal models that FHF significantly alleviates MPN progression, including EPO-induced polycythemia vera (PV)-like, JAK2^V617F^-driven PV, and MPL^W515L^-driven essential thrombocythemia (ET) models. FHF effectively reduced erythrocyte aggregation-induced thrombosis in PV and reversed myelofibrosis in ET. To explore the therapeutic effects, active components, and mechanisms of FHF in MPNs, we performed network pharmacology and RNA-seq analyses, with subsequent experimental validation. The results indicate that the therapeutic benefits of FHF are closely associated with cellular senescence and inflammatory responses. Validation experiments showed that FHF induces cellular senescence via activation of the p53/p21 pathway and suppresses inflammation by inhibiting the STAT3 and NF-κB signaling pathways. Our study provides scientific evidence supporting the use of FHF in the treatment of MPNs.

## Highlights

• FHF treatment alleviated the disease burden and progression in multiple mouse models of MPNs.
• FHF inhibits MPN cell proliferation by inducing senescence via activating p53/p21 signaling pathway.
• FHF improves the progression of MPNs by inhibiting the STAT3 and NF-κB signaling pathways.

## Graphical abstract

**Figure:**
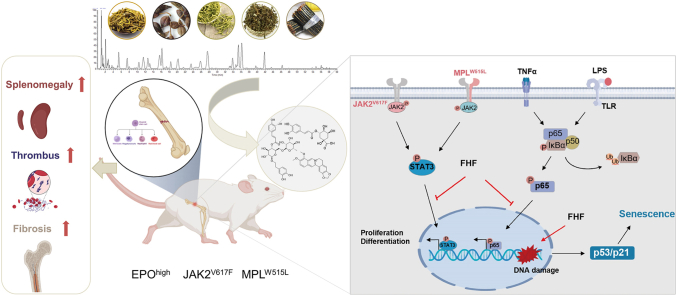

## 1 Introduction

Myeloproliferative neoplasms (MPNs) are chronic blood cancers marked by the overproduction of myeloid lineage cells in the bone marrow [1], which increases the risk of thrombosis and leukemia transformation [2,3]. MPNs are classified into three main subtypes according to their clinical manifestations, including polycythemia vera (PV), marked primarily by erythrocyte overproduction, essential thrombocythemia (ET), characterized mainly by platelet excess, and primary myelofibrosis (PMF), distinguished by pathological marrow fibrosis and scarring [2,4]. Approximately 95% of all MPNs are caused by mutations in the Janus-associated kinase 2 (JAK2) gene, the myeloproliferative leukemia virus proto-oncogene (MPL) gene, or the calreticulin (CALR) gene. These mutations lead to persistent activation of the JAK-STAT signaling pathway [5]. Despite the availability of JAK inhibitors such as ruxolitinib, current therapies for MPN remain limited by adverse effects, incomplete disease modification, and the lack of agents that simultaneously target both malignant cell proliferation and the inflammatory microenvironment. MPN patients exhibit increased levels of pro-inflammatory cytokines in their serum, including C-X-C motif chemokine ligand 10 (CXCL10), interleukin (IL)-6, tumor necrosis factor α (TNF-α), IL-1β, and IL-15 [[6], [7], [8]]. A mounting body of evidence indicates that persistent inflammation plays a pivotal role in the disease progression and transformation of MPNs into acute leukemia [[9], [10], [11], [12], [13], [14]]. NF-κB, a universal transcription factor that controls numerous inflammatory and immune-related genes, has been demonstrated as a key signaling node in malignant cells in MPNs [15,16]. The continuous production of TNF-α, a hallmark of MPNs resulting from impaired suppression of Toll-like receptor (TLR) signaling, has been shown to promote malignant clone proliferation in MPNs [17,18]. This finding was further corroborated by the therapeutic effect of the TNF receptor antibody, which alleviated the myeloproliferative phenotypes in the JAK2^V617F^-driven MPN mouse model [19]. Aberrant activation of the STAT3 signaling pathway is a key step in driving the continuous amplification of inflammatory responses [[20], [21], [22], [23]]. By regulating the expression of multiple pro-inflammatory factors, STAT3 participates in and maintains the chronic inflammatory state of the MPN microenvironment, thereby promoting disease progression [24]. A number of studies have demonstrated the efficacy of analogous therapeutic interventions that target inflammatory pathways in the reduction of MPN burden and the mitigation of its progression [16,[25], [26], [27]].

Traditional Chinese medicine offers the advantages of multi-targeting and low toxicity in disease treatment. Fufang Huangbo Formula (FHF) is a traditional Chinese medicine composed of a combination of *Phellodendri Chinensis Cortex*, *Forsythiae Fructus*, *Taraxaci Herba*, *Lonicerae Japonicae Flos*, and *Scolopendra*. FHF was originally used to treat osteomyelitis. It has now demonstrated efficacy in the clinical treatment of inflammatory disorders, including skin ulcers, ulcerative colitis, diabetes ulcers, seborrheic dermatitis [28,29]. It has been proven to accelerate wound repair in diabetic through Nrf2 signaling [29]. FHF reduces IL-6 and TNF-α production by inhibiting the activation of the NF-κB/COX-2 signaling pathway in ulcerative colitis [30]. Network pharmacological analysis and experimental validation have shown that the formula's main component, phellodendrine, promotes cellular autophagy by modulating the AMPK/mTOR signaling pathway, thereby reducing gut damage in ulcerative colitis [28].

In conclusion, current MPN treatments have limitations such as side effects and incomplete remission. FHF shows anti-inflammatory efficacy but its effects on MPN are unknown. This study used multiple MPN models, network pharmacology, RNA-seq, and validation experiments to explore FHF's efficacy and mechanism, providing a new strategy for MPN treatment.

## 2 Materials and methods

### 2.1 Reagents and plasmids

The FHF preparation was prepared according to the *Chinese Pharmacopoeia* by combining the five constituent herbs at specified mass ratios and following standard manufacturing procedures [31]. Quality control was performed according to *Chinese Pharmacopoeia* standards, with phillyrin and berberine hydrochloride employed as reference markers. The detailed preparation process is as follows: 40 g of *Phellodendri Chinensis Cortex*, 80 g of *Forsythiae Fructus*, 40 g of *Taraxaci Herba*, 40 g of *Lonicerae Japonicae Flos*, and 2.4 g of *Scolopendra* were extracted three times with water. The combined extracts were filtered, and ethanol was added to achieve a final concentration of 70% (*v*/*v*). After standing for 24 h, the mixture was filtered again, and the filtrate was concentrated under reduced pressure to remove ethanol. The resulting residue was then dissolved in water. The dosage of 0.5 g/kg/day was selected based on the results of our preliminary experiments. In the pre-experiment, three doses (0.25, 0.5, and 1.0 g/kg/day) were administered to mice for two consecutive weeks. No signs of toxicity or adverse effects were observed in the 0.25 and 0.5 g/kg/day groups, whereas mild body weight loss was noted in the 1.0 g/kg/day group. Consequently, the 0.5 g/kg/day dose was chosen for subsequent animal experiments. Ruxolitinib was purchased from TargertMOI (Cat T1829). MSCV-MPL-IRES-EGFP and MSCV-MPL^W515L^-IRES-EGFP plasmids were obtained from the preliminary construction of our laboratory, and verified by sequencing.

### 2.2 UHPLC-Q-Orbitrap conditions

UHPLC-Q-Orbitrap-HRMS (Waltham, MA, USA) was used to analyze the composition of FHF. Chromatographic separation was performed using an UPLC BEH Shield RP18 column (2.1 × 100 mm, 1.7 μm). The mobile phases consisted of water with 0.1% formic acid (phase A) and methanol with 0.1% formic acid (phase B). Phase B mobile proportions were as follows: 5%-20% (0-47 min); 20%-30% (47-54 min), 30%-80% (54-60 min). The final data was analyzed using Thermo Xcalibur 4.0 software.

### 2.3 Network pharmacology analysis

Target prediction of chemical components in FHF was performed using SwissTargetPrediction (https://swisstargetprediction.ch/). Screening of genes involved in MPNs was conducted by DrugBank (https://go.drugbank.com/), DisGeNET (https://disgenet.com/), GeneCards (https://www.genecards.org/), TTD (https://ttd.idrblab.cn/), and OMIM (https://www.omim.org/) databases. Protein-protein interaction analysis was performed by STRING website (https://cn.string-db.org/, version 11.0). GO and KEGG enrichment analyses were performed using the Metascape database (https://metascape.org/gp/version 3.5.20230101). Cytoscape software (version 3.9.1) and Sangerbox website (http://sangerbox.com/) were used for visualization.

### 2.4 Animal experiments

All animal studies were performed in accordance with the Guidelines for the Care and Use of Laboratory Animals and were approved by the Institutional Animal Care and Use Committees at Shandong University. C57BL/6J and BALB/C mice purchased from PengYue (Jinan, China). JAK2^V617F^-floxed mice and Vav-Cre mice were obtained from the Jackson Laboratory. The design and performing of animal experiments were approved by the Institutional Animal Care and Use Committees at Shandong University. Approval number: KYLL-2023(ZM)-576.

For EPO^high^-induced PV-like model, C57BL/6J mice were intraperitoneally injected with EPO (5000 UI/kg) every 2 days during 3 weeks [32]. The mice were then randomly allocated to different groups of six each receiving placebo (PBS, i.p.) or FHF (0.5 g/kg/day, i.p.). JAK2^V617F^-floxed mice (C57BL/6J background) were crossed with Vav-Cre mice (C57BL/6J background) to generate JAK2^V617F^ knock-in (JAK2^V617F^ KI) mice. We obtained bone marrow cells from JAK2^V617F^ KI mice and transplanted them into sub-lethal dose irradiated (550 cGy) recipient mice. The recipient mice developed MPN-like disease features of leukocytosis and erythrocyte within 2 weeks, were randomised into groups of six mice each, and underwent drug administration as above (PBS or FHF, 0.5 g/kg/day, i.p.). For the MPL^W515L^-induced MPN model, c-Kit^+^ bone marrow cells from 8-week-old BALB/c mice were stimulated with cytokines and transduced twice with the MSCV-MPL-IRES-EGFP or MSCV-MPL^W515L^-IRES-EGFP retrovirus, followed by tail vein injection into irradiated (600 cGy) recipient BALB/c mice. The recipient mice developed ET-like disease features of leukocytosis and thrombocytosis within 2 weeks, were randomised into groups of six mice each, and underwent drug administration as above (PBS or FHF, 0.5 g/kg/day, i.p.).

### 2.5 Cell culture

HEK293T cells were purchased from the Cell Bank of the Shanghai Institute for Biological Sciences, Chinese Academy of Science, and were cultured in DMEM medium (BasalMedia, L110KJ) supplemented with 10% FBS (SeraPure, SE141-500). HEL cells were cultured in RPMI 1640 medium supplemented with 10% FBS and 100 mg/mL penicillin-streptomycin mix. SET-2 cells were maintained in RPMI 1640 medium with 20% FBS plus 100 mg/mL penicillin-streptomycin mix.

### 2.6 Flow cytometry assays

Single cell suspensions of bone marrow, spleen or cell lines were performed as previously described [33]. Detailed information on the antibodies used in this study is shown in Table S4 in the Supporting Information.

### 2.7 Colony-forming assay

Bone marrow lineage negative cells were obtained using the isolation kit (STEMCELL, #19856) and plated in the corresponding methylcellulose medium. For CFU-GM, 1.8 × 10^4^ lineage negative cells were cultured in MethoCult™ M3534 methylcellulose medium, followed by colony counting at day 7. For CFU-GEMM, 1.8 × 10^4^ lineage negative cells were plated in MethoCult™ M3234 enriched with 10 ng/mL IL-3, 3 U/mL EPO, 10 ng/mL IL-6, and 50 ng/mL SCF, followed by colony counting after 7 days.

### 2.8 Cell proliferation assay

Cells were plated in 96-well plates at a density of 4000 cells/well, and exposed to DMSO, FHF (20 μg/mL, 30 μg/mL, and 40 μg/mL), or Ruxolitinib (2.5 μM) for 72 h. Cell proliferation was measured using a Cell Counting Kit-8 (bimake B34302). The EdU assay kit (RiBoBio C10310-1) was used to determine the frequency of cells with active DNA replication, which represents cell proliferation status, following the manufacturer's protocol.

### 2.9 SA-β-Gal staining

MPN cells were exposed to doxorubicin and FHF for 24 h and then assessed for senescence using the SA-β-Gal staining kit (Beyotime C0602). Senescence rates were determined by microscopic evaluation of SA-β-Gal-stained cells, with quantification based on positive cell percentages in randomly sampled fields.

### 2.10 Real-time qPCR analysis

Total RNA extraction and cDNA preparation was performed as previously described [34]. RNA expression levels were analyzed by the 2^−ΔΔCT^ method (18S-normalized), with all quantitative PCR primers detailed in Supplemental Table S5.

### 2.11 RNA-sequencing

SET-2 cells were collected after FHF treatment for 18 h. RNA isolation was performed using TRIzol reagent. RNA sequencing was performed using the ST-E00205 platform. Data processing and differential expression analysis were performed using DESeq2 (version 1.32.0) with R (version 4.1.2). GSEA was performed using GSEA software (version 4.2.3). RNA-seq data that support the findings is openly available in the NCBI GEO database (GSE252715).

### 2.12 Luciferase reporter assay

A luciferase reporter plasmid carrying the NF-κB element was transfected into SET-2 cells for 24 h. Cells were co-treated with FHF and LPS (2 μg/mL) for 16 h, and luciferase activity was measured using the Dual Luciferase Reporter Assay System (Promega E1910).

### 2.13 Immunoblotting

Protein extraction was carried out in RIPA buffer with a protease/phosphatase inhibitor cocktail, followed by SDS-PAGE separation, PVDF membrane transfer, and 5% BSA blocking. The protein levels were determined by incubation with primary antibodies (Table S4).

### 2.14 Blood count

Peripheral blood of mice was collected in EDTA-treated blood collection tubes, and blood was detected by hematology analyzer within 30 min.

### 2.15 Patient samples

Peripheral blood samples from MPN patients and healthy donors were obtained at the Heze Municipal Hospital in China. CD34^+^ cells were enriched by using the CD34 Positive Selection Kit II (STEMCELL, 17856). 1000 CD34^+^ cells were seeded per well in MethoCult™ H4434 methylcellulose medium containing cytokine supplements with co-cultured FHF. The number of colonies was counted on day 14.

### 2.16 Apoptosis and cell-cycle analysis

For apoptosis analysis, the cells were cultured with FHF for 24 h, followed by 15 min staining with Annexin V (Biolegend, 640920), and added propidium iodide (PI, 50 μg/mL), with subsequent flow cytometry analysis.

For cell cycle, cells were cultured with FHF for 24 h, followed by overnight fixation in 70% ethanol at −20 °C. Following PBS washes, cells were incubated with PI at 37 °C for 30 min, then analyzed for cell cycle phases via flow cytometry. For G0 and G1 cells, cells were stained with LIVE/DEAD™ (Thermo L34968) for 30 min at room temperature. The cells were then washed twice with PBS, and stained with Ki67 (eBioscience, 11-5698-82) and Hoechst33342 (10 μg/mL) for 1 h, followed by flow cytometry analysis.

### 2.17 Immunofluorescence staining

Cells were fixed in 4% paraformaldehyde. Then, the cells were permeabilized with 0.1% Triton X-100 solution. After 1 h blocking in 3% BSA, cells were exposed to primary antibodies for overnight incubation at 4 °C. The next day, cells were incubated with the fluorescent labeled Alexa Fluor 488-conjugated secondary antibody for 1 h. After the cells were washed three times (keep in the dark), DAPI staining was performed for 5 min. The images were obtained using a fluorescence microscope (Olympus BX53).

### 2.18 Statistical analysis

Data analysis was conducted using Prism 9 with unpaired two-tailed Student's *t*-test, unless otherwise indicated. For the survival data, *P* values were determined by the Log-rank (Mantel-Cox) test. For the cell proliferation rate, the *P* value was determined by two-way ANOVA. For the two-way ANOVA, normality was assessed using the Shapiro–Wilk test and homogeneity of variances was confirmed with Levene's test. No data transformations were applied, as all datasets satisfied the required assumptions (*P* > 0.05 for both tests). Results are expressed as mean ± SEM, with *P* values < 0.05 considered significant.

## 3 Results

### 3.1 Chemical composition analysis of FHF

**Fig. 1:**
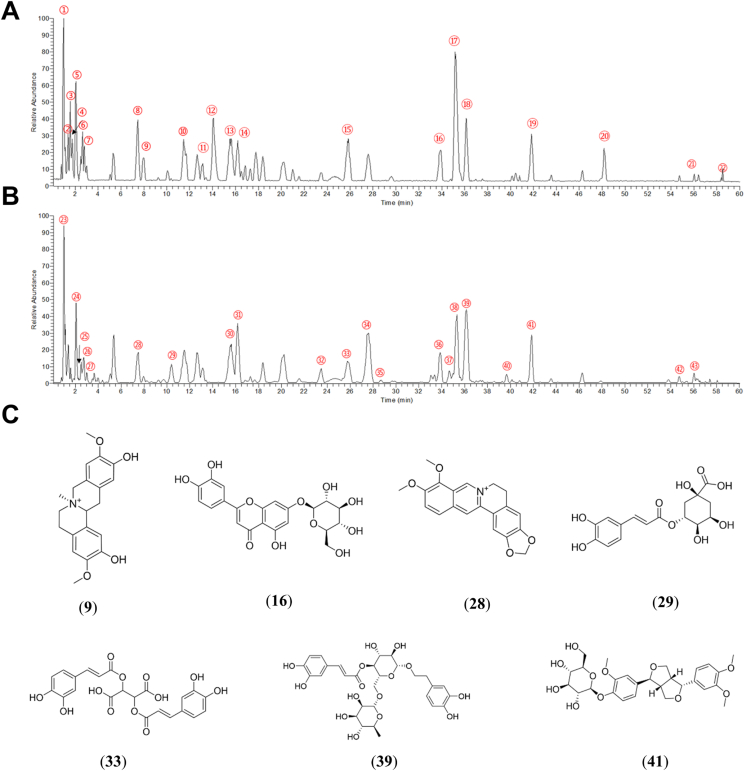
Identification of the chemical constituents of FHF by UHPLC-MS/MS. (A) The total ion chromatogram of the FHF sample in positive ion modes. (B) The total ion chromatogram of the FHF sample in negative ion modes. (C) Representative chemical composition Formula for FHF identification. (**9**) phellodendrine; (**16**) luteolin-7-*O*-glucoside; (**28**) berberine; (**29**) chlorogenic acid; (**33**) chicoric acid; (**39**) forsythiaside A; (**41**) phillyrin.

UHPLC-MS/MS was performed for chemical composition analysis and identification of major compounds in FHF. Chemical analysis identified 43 constituents, comprising 9 alkaloids, 9 phenolic acids, 9 phenylpropanoids, 4 terpenoids, 3 flavonoids, 1 alkyne, and 8 others (Fig. 1A–C and Table S1). Berberine (**28**) and phellodendrine (**9**) are the main ingredients of *Phellodendri Chinensis Cortex* in the Formula. Forsythiaside A (**39**) and phillyrin (**41**) are the main constituents of *Forsythiae Fructus* in the Formula. Chlorogenic acid (**29**) and luteolin-7-*O*-glucoside (**16**) are the major constituents of *Lonicerae Japonicae Flos*, while chicoric acid (**33**) is the main constituent of *Taraxaci Herba*. The identification of chemical components in FHF can serve as a foundation for quality control. Furthermore, according to the *Chinese Pharmacopoeia*, the contents of berberine and phillyrin are utilized as a quality control standard for FHF. Consequently, we employed HPLC to ascertain the contents of berberine and phillyrin in FHF. The results demonstrated that the content of berberine and phillyrin complies with the standards of the Chinese Pharmacopoeia (Fig. S1A–C).

### 3.2 FHF treatment ameliorated EPO^high^-induced PV-like MPN mice

To assess safety, we first performed acute toxicity tests on FHF. No hematologic abnormalities were observed in the peripheral blood of mice after 21 days of FHF treatment (Fig. S2A). The body weight and spleen size of the FHF-treated mice were comparable to those of the placebo control group. (Fig. S2B–D). Histological analysis results showed that FHF did not cause significant toxic reactions in various organs, including the spleen, lungs, kidneys, and liver (Fig. S2E). The above results indicate that FHF is safe and non-toxic for *in vivo* administration.

**Fig. 2:**
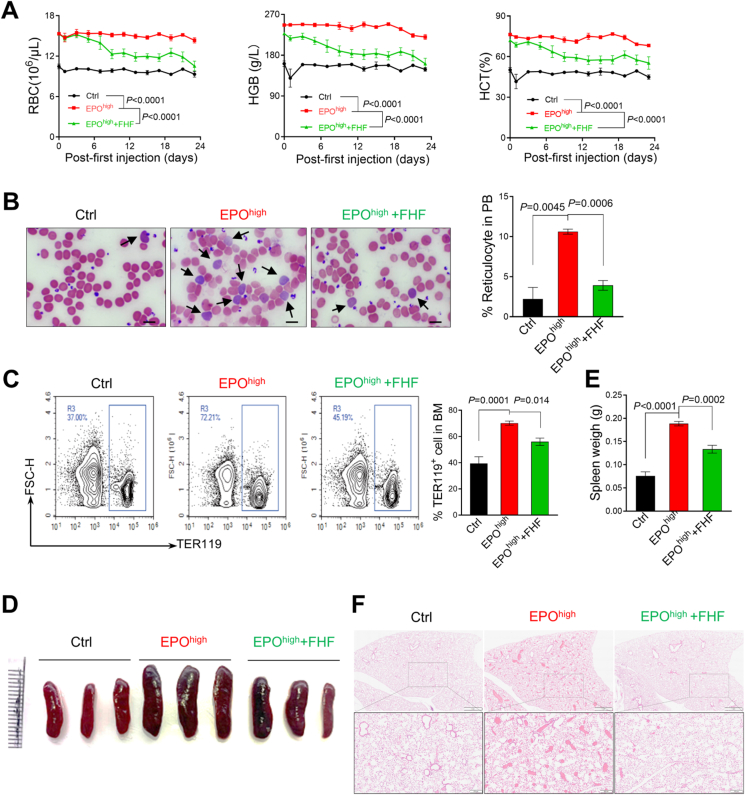
FHF treatment ameliorated EPO^high^-induced PV-like MPN mice. (A) RBC, HGB, and HCT in peripheral blood from indicated mice after treatment. N = 6 mice per group. Ctrl represents the control mice with equal placebo (PBS) injection. (B) Peripheral blood smears from treated mice after 23 days. Quantification of reticulocytes was shown on the right. Scale bar: 50 μm. (C) Analysis of TER119^+^ cell populations using flow cytometry in bone marrow. (D) Representative spleen from indicated group mice. (E) Statistical analysis of spleen weights. (F) Representative images of mouse lung tissue stained with H&E.

To evaluate the therapeutic potential of treating MPNs, the EPO^high^-induced PV-like mouse model was established by subjecting mice to repeated high doses of EPO (5000 U/kg/2 days). As previously reported [35], the mice showed obvious PV-like phenotypes, indicated by the increased red blood cells (RBC), hematocrit (HCT), and hemoglobin (HGB) levels in peripheral blood (Fig. 2A). Reticulocytes were also largely increased in the peripheral blood, reflecting erythroid hyperproliferation (Fig. 2B). The FHF treatment significantly reduced the elevated erythrocytosis, almost returning it to normal levels by day 23 of administration in the control group (Fig. 2A and B). A similar reduction in erythroblasts was observed in the bone marrow of FHF-treated EPO^high^ mice compared to the placebo-treated group (Fig. 2C). Furthermore, FHF administration significantly reduced the size and weight of the spleen in EPO^high^-induced PV-like mice (Fig. 2D and E).

Excessive production of RBCs leads to the abnormal accumulation of blood cells, forming embolus-like blood masses in various organs of PV mice [35]. Histological analysis demonstrated that the pulmonary blood masses in lungs of EPO^high^-induced PV-like mice were completely covered by treatment with FHF (Fig. 2F). These findings indicated that FHF treatment ameliorated the progression of the disease in EPO^high^-induced PV-like mice.

### 3.3 FHF provided therapeutic benefit in JAK2^V617F^-induced MPN mice

**Fig. 3:**
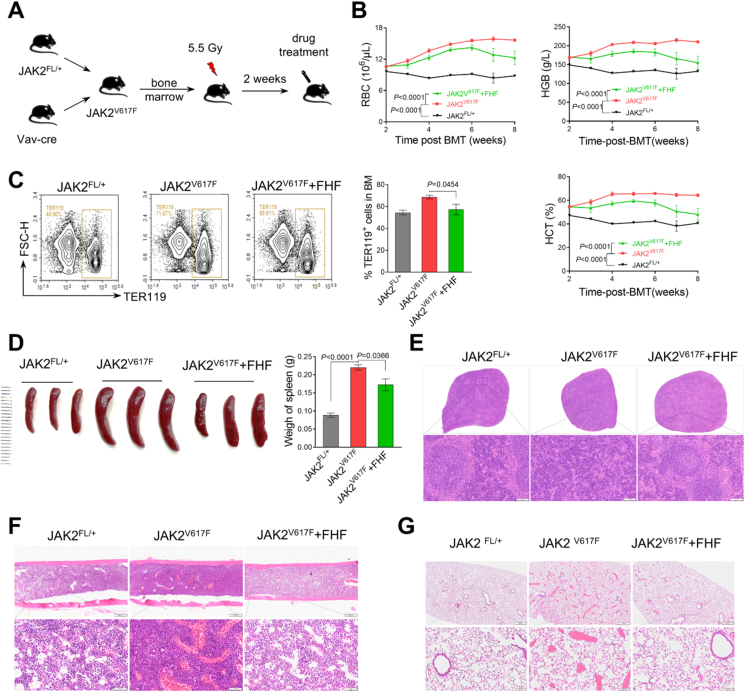
FHF provided therapeutic benefit in JAK2^V617F^-induced MPN mice. (A) Schematic diagram of the JAK2^V617F^-driven MPN mice and therapeutic intervention. Transplantation donors of bone marrow cells expressing JAK2^V617F^ were treated with vehicle or FHF for 6 weeks. (B) RBC, HGB, and HCT counts in PB from indicated mice after treatment. N = 6 mice per group. (C) Results of flow cytometry analysis of TER119^+^ cells in bone marrow. (D) Spleen size and weight of indicated mice after FHF treatment. (E-G) Representative H&E staining of spleen, BM and lung.

To further confirm the effects of FHF on MPNs, we next tested the therapeutic response of FHF in a JAK2^V617F^-induced MPN model [36]. Total bone marrow cells from JAK2^V617F^ KI mice were transplanted into sub-lethal dose irradiated recipient mice (Fig. 3A), in which obvious polycythemia and mild leukocytosis were developed after two weeks of bone marrow transplantation (Fig. S3A). The recipient mice were treated with FHF via intraperitoneal injection twice daily for 6 weeks. The data demonstrated that FHF treatment significantly reduced the levels of RBC, HGB, and HCT in the peripheral blood (Fig. 3B), and decreased the elevated erythroblasts in the bone marrow (Fig. 3C). Indeed, FHF treatment largely normalized the spleen weight, bone marrow, and splenic structure in mice bearing JAK2^V617F^ (Fig. 3D–F). FHF also significantly reduced the formation of blood clots resulting from excessive RBC aggregation in various organs, including the lungs and liver from the mice transplanted with JAK2^V617F^ bone marrow (Fig. 3G and Fig. S3B).

### 3.4 FHF significantly alleviated the progression of the disease in MPL^W515L^-driven MPN mice

**Fig. 4:**
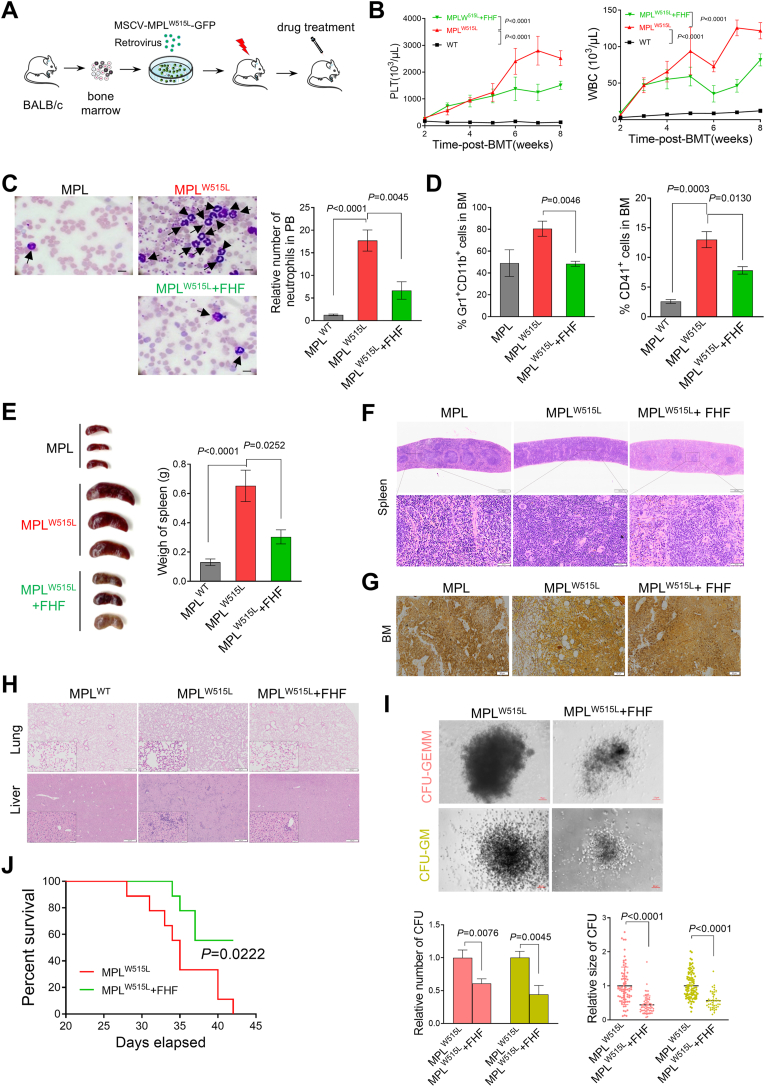
FHF alleviated the progression of the disease in MPL^W515L^-driven MPN mice. (A) Strategy diagram of MPL^W515L^-driven ET mouse model establishment and drug treatment. (B) PLT (left) and WBC (right) counts in peripheral blood from indicated group mice after treatment. N = 6 mice per group. (C) Representative peripheral blood smear of indicated group of mice. Quantification of neutrophils in peripheral blood smear was shown on the right. Scale bar: 50 μm. (D) Flow cytometry analysis of myeloid cells (CD11b^+^Gr1^+^) and megakaryocytes (CD41^+^) after FHF or placebo treatment in bone marrow. (E) Spleen size and weight of indicated mice after FHF or placebo treatment. (F) Representative H&E staining of bone marrow from indicated group mice. (G) Representative fibrotic staining of the bone marrow reticulum. (H) Representative H&E staining of lung. (I) CFU-GEMM and CFU-GM colony formation assays of lineage negative cells after FHF or placebo treatment. Colony number and size were quantified. (J) Kaplan-Meier survival curves. N = 9 mice for each group. *P* value was determined by a Log-rank test.

Next, we evaluated the effects of FHF on a well-established MPN model driven by MPL^W515L^ mutation. Transduction of bone marrow hematopoietic stem progenitor cells (HSPC) with a retrovirus encoding MPL^W515L^, which was followed by transplantation into irradiated BALB/c mice (Fig. 4A). As expected, thrombocytosis and leukocytosis were observed in MPL^W515L^ recipient mice after two weeks of transplantation (Fig. S4A). MPL^W515L^ recipient mice were treated with placebo or FHF for 6 weeks. FHF significantly reduced elevated white blood cell (WBC) and platelet (PLT) counts in MPL^W515L^ recipient mice (Fig. 4B). This finding was further confirmed by the largely reduced frequency of neutrophils in the peripheral blood of MPL^W515L^ recipient mice (Fig. 4C). Flow cytometry analysis showed that myeloid cells (Gr1^+^Mac1^+^) and megakaryocytes (CD41^+^) in bone marrow of MPL^W515L^ recipient mice were also significantly reduced after FHF treatment, indicating that FHF markedly reduced the megakaryopoiesis and leukocytosis driven by MPL^W515L^ (Fig. S4B and Fig. 4D).

MPN mouse models were also characterized by the extramedullary hematopoiesis resulting in the splenomegaly, and eventually myelofibrosis due to the abnormal hematopoiesis in the bone marrow. Notably, compared with the placebo-treated mice, FHF treatment significantly alleviated splenomegaly in MPL^W515L^ recipient mice (Fig. 4E). This was accompanied by the obvious normalization of the splenic architecture following treatment with FHF. (Fig. 4F). Bone marrow histology showed a significant reduction in fibrosis in MPL^W515L^ recipient mice treated with FHF (Fig. 4G). Moreover, we observed a significant reduction in blood cell migration to the liver and lungs compared to the placebo group (Fig. 4H).

We performed colony formation assays with bone marrow lineage negative cells to assess the effects of FHF on HSPC expressing MPL^W515L^. MPL^W515L^ mice treated with FHF showed a significantly reduced number and size of GEMM and GM colony formation compared to placebo-treated groups. (Fig. 4I). We also performed a secondary transplantation with bone marrow from the first MPL^W515L^ recipient mice, followed by the treatment of FHF or placebo on day 21. All placebo-treated mice died on day 42, while 60% of the FHF-treated group survived, indicating that FHF significantly extended the survival of MPL^W515L^ recipient mice (Fig. 4J).

### 3.5 Network pharmacology analysis of the mechanism of FHF in treating MPNs

**Fig. 5:**
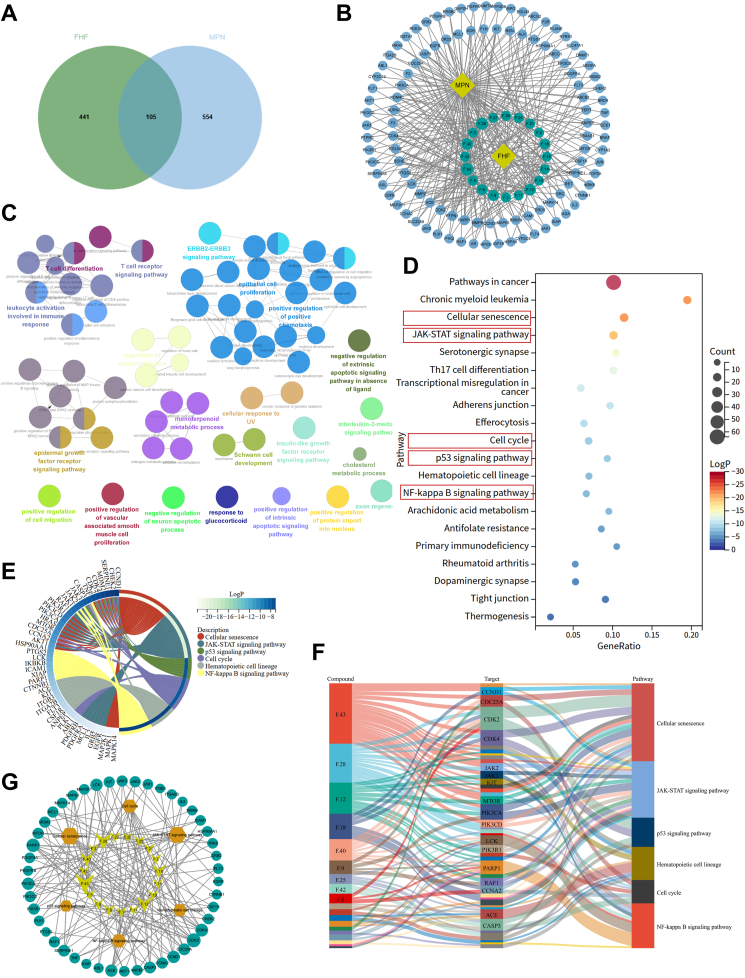
Network pharmacological analysis of FHF treatment for MPNs. (A) The intersection targets the active targets of FHF and the MPN-related targets. (B) Herb-ingredient-target-disease network in the treatment of FHF on MPNs. (C) ClueGO enrichment analysis in Cytoscape was used to analyze the 105 common targets of the biological processes of GO. (D) Bubble plot shows KEGG signaling pathways enrichment analysis of 105 common targets. (E) Chordal plot shows the main targets-pathways. (F) Sankey diagram shows the main component-target-signaling pathway diagram of FHF against MPNs. (G) Interaction network diagram of key signaling pathway-target-major active components.

Network pharmacology was used to analyze the potential components, targets, and molecular mechanisms of FHF for treating MPNs. A total of 105 targets for FHF to intervene in MPNs were ultimately identified by conducting a cross-analysis of the disease targets of MPNs and the action targets of FHF (Fig. 5A). Network analysis was performed on the key targets of the FHF intervention in MPN and their corresponding active components to visualize the complex associations between these components and potential targets (Fig. 5B). GO enrichment analysis revealed that the biological processes primarily involved leukocyte activation in the immune response, epithelial cell proliferation, and the epidermal growth factor receptor signaling pathway (Fig. 5C). KEGG signaling pathway analysis showed that the immune-related signaling pathways primarily involved were mainly the cellular senescence, the JAK-STAT signaling pathway, the p53 signaling pathway, hematopoietic cell lineage, and the NF-κB signaling pathway (Fig. 5D and E). Additionally, based on a reverse strategy, the primary active components acting on these signaling pathways were identified as alkaloids (e.g. berberine, isocorypalmine, phellodendrine) (Fig. 5F and G, Table S2). In conclusion, network pharmacology suggests that the intervention of FHF in MPNs is related to molecular mechanisms involving cellular senescence, the JAK-STAT signaling pathway, and the NF-κB inflammatory signaling pathway. Based on the above enrichment analysis results and their relevance to the known pathogenesis of MPNs, we subsequently performed experimental validation on the cellular senescence, JAK-STAT, and NF-κB signaling pathways.

### 3.6 FHF inhibited the proliferation of MPN cells via cell senescence

**Fig. 6:**
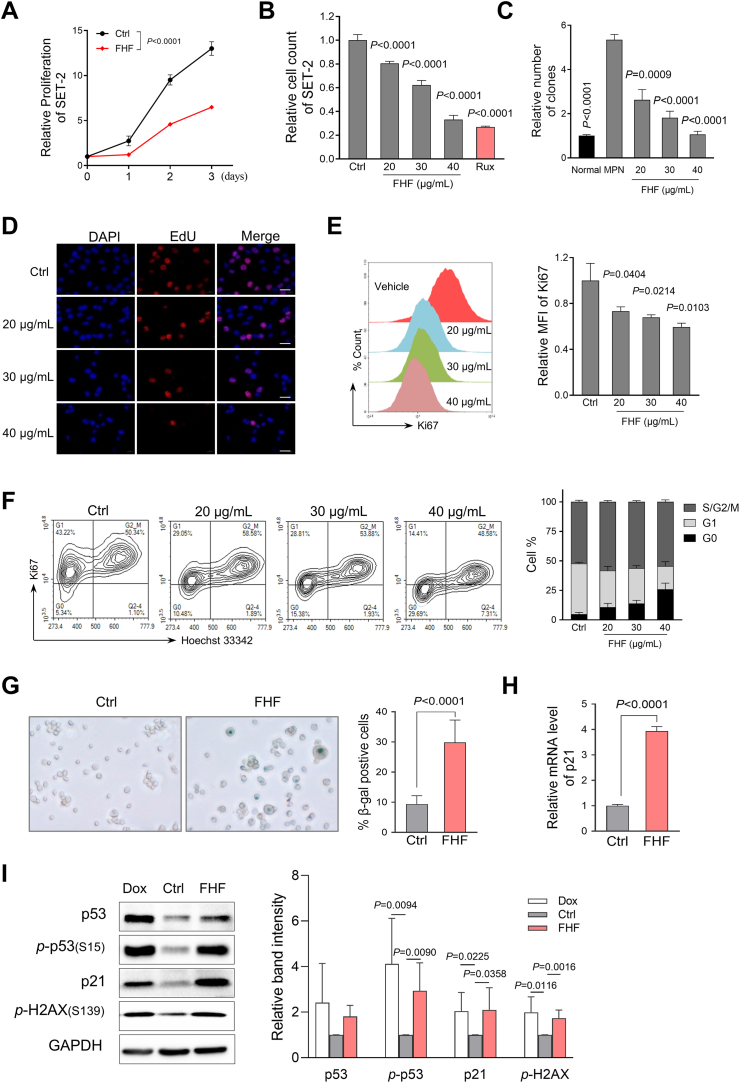
FHF inhibited the proliferation of MPN cells by inducing senescence. (A) The effect of FHF (30 μg/mL) treatment at different time points on SET-2 cell proliferation. Ctrl represents vehicle control. (B) Effect of treatment with different FHF concentrations for 24 h on the proliferation of SET-2 cells. Rux was used as a positive control. (C) Primary MPN patients' CD34^+^ cells with FHF treatment were analyzed in colony-forming unit assays for 14 days. Colony number was quantified. Normal represents healthy donor control. (D) Representative EdU staining images of SET-2 cells after 48 h with FHF treatment. Scale bar: 20 μm. (E) Ki67 levels in SET-2 cells treated with FHF for 24 h were measured by flow cytometry. The quantitative result was shown on the right. (F) Cell cycle analysis by flow cytometry in SET-2 cells with FHF treatment for 24 h. (G) Representative images and statistical analysis of SA-β-gal staining of SET-2 cells treated with FHF for 24 h. (H) Quantitative PCR analysis of p21 levels with FHF treatment for 12 h in SET-2 cells. (I) Immunoblotting analysis of indicated proteins in SET-2 cells treated by FHF (30 μg/mL) for 24 h. Dox (0.3 μM) was used as positive control.

Considering the above findings that FHF significantly reduced disease burden in MPN mouse models, we evaluated the effect of FHF on MPN cell proliferation. FHF treatment significantly inhibited the proliferation of both SET-2 and HEL cells harboring the JAK2^V617F^ mutation (Fig. 6A and B and Fig. S5A and B). Notably, we also observed a dose-dependent reduction in colony-forming ability of CD34^+^ cells obtained from JAK2^V617F^-positive MPN patients after FHF treatment (Fig. 6C).

EdU assays demonstrated that FHF largely suppressed the DNA replication in SET-2 cells (Fig. 6D). Consistent with this, Ki67 expression, a well-known marker of active cell proliferation, also showed a dose-dependent reduction in FHF-treated SET-2 cells (Fig. 6E). We also measured the cell viability and found that FHF only caused apoptosis at high doses. This indicates that apoptosis is not the primary reason for FHF-induced inhibition of cell proliferation (Fig. S5C).

To order to further clarify the effects of FHF on SET-2 cell proliferation, cell cycle analysis was performed using Ki67/Hoechst33342 staining, and found that FHF treatment led to a significant increase in the frequency of cells at G_0_ phase (Fig. 6F). SA-β-gal (a biomarker of senescence) staining showed that the percentage of senescence cells was remarkably increased in SET-2 cells with FHF (Fig. 6G). FHF increased p21 expression and the phosphorylation of H2AX and p53 (Fig. 6H and I). Similar findings were also observed in HEL cells following treatment with FHF (Fig. S5D–E). In summary, consistent with the findings of network pharmacology, FHF inhibits the proliferation of MPN cells by activating the p53/p21 signaling pathway and promoting cell senescence.

### 3.7 FHF regulated STAT3 and NF-κB signaling pathways in MPN cells

**Fig. 7:**
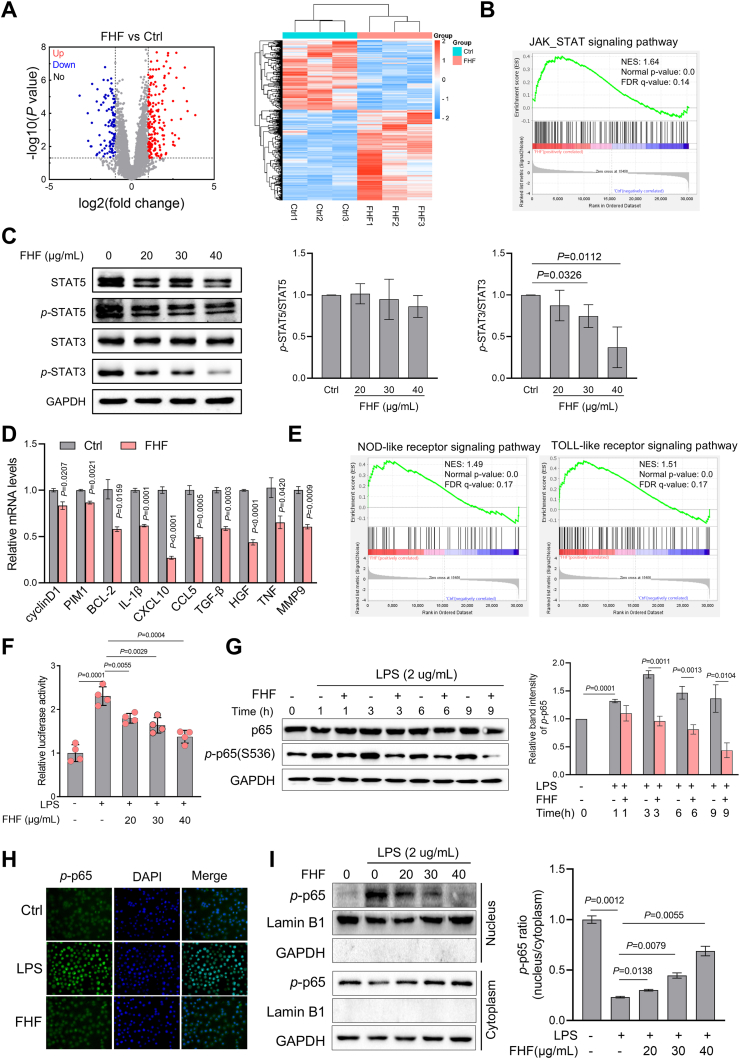
FHF regulated STAT3 and NF-κB signaling in MPN cells. (A) Volcano plot and heatmap visualization of differential gene expression in SET-2 cells treated by FHF (30 μg/mL) for 18 h. Fold Change ≥2, *P* < 0.05. (B) GSEA of JAK/STAT signaling pathway in SET-2 cells treated by FHF. (C) Immunoblotting analysis of indicated proteins in SET-2 cells treated by FHF for 24 h. (D) Quantitative PCR analysis of STAT3 target genes in SET-2 cells treated by FHF (30 μg/mL). (E) GSEA of NOD-like and TOLL-like receptor signaling pathways in SET-2 cells treated by FHF. (F) Luciferase reporter assays of NF-κB-response element in SET-2 cells with FHF treatment upon 2 μg/mL LPS induction. (G) Immunoblotting analysis of p65 and *p*-p65 in SET-2 cells treated by FHF (30 μg/mL) upon 2 μg/mL LPS induction. (H) Immunofluorescence assessment of FHF effects on *p*-p65 nuclear localization in SET-2 cells. (I) Immunoblotting analysis of *p*-p65 expression in the nucleus and cytoplasm in SET2 cells treated by FHF (30 μg/mL). LPS: lipopolysaccharide; GSEA: gene set enrichment analysis.

To gain insight into the molecular mechanism of FHF on MPN cells, RNA sequencing was performed on FHF-treated SET-2 cells. A total of 296 differentially expressed genes (DEG) were identified in SET-2 cells following treatment with FHF (Fig. 7A). Consistent with the finding that FHF inhibits SET-2 cell proliferation, gene set enrichment analysis (GSEA) revealed that these transcriptional changes are significantly associated with p53 signaling, cell cycle regulation, and DNA replication (Fig. S6A–C and Table S3). Notably, the JAK/STAT signaling pathway was also significantly enriched (Fig. 7B). Immunoblotting showed that FHF treatment reduced the phosphorylation of STAT3, but had no apparent effect on the phosphorylation of STAT5 (Fig. 7C). This was further confirmed by the decreased expression of STAT3 target genes after FHF treatment, including BCL-2, CCL5, IL-1β, TGF-β, and TNF (Fig. 7D).

Additionally, genes associated with immune response were also markedly altered in SET-2 cells after FHF treatment, including TOLL-like and NOD-like receptor signaling pathways that ultimately activate NF-κB signaling (Fig. 7E). To confirm the effect of FHF on this signaling pathway, we examined the activity of NF-κB in SET-2, and found that FHF significantly reduced the expression of inflammatory factors induced by lipopolysaccharide (LPS), including CCL2, IL-16, IL-18, and MMP9 (Fig. S6D). We investigated whether FHF primarily exerts its regulatory effect by modulating NF-κB. The results of the luciferase activity experiment show that LPS substantially stimulated the transcriptional activity of NF-κB, which was significantly reduced by FHF in a concentration-dependent manner (Fig. 7F). Immunoblotting results showed that FHF significantly inhibited the phosphorylation of p65 in SET-2 cells (Fig. 7G). The immunofluorescence results showed that the nuclear translocation of p-p65 was significantly inhibited (Fig. 7H and I). These data indicate that FHF regulates the proliferation and inflammatory response of MPN cells by inhibiting the NF-κB and STAT3 signaling pathways.

### 3.8 Molecular docking analysis of key active ingredients

**Fig. 8:**
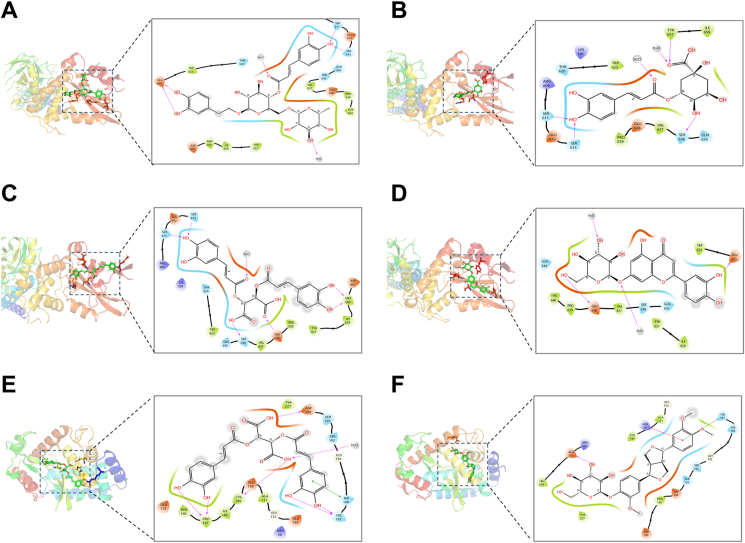
Docking patterns of the core target, STAT3 and NF-κB proteins, with the major active compounds in the FHF anti-MPN treatment. (A) Molecular docking of STAT3 with forsythiaside A (**39**). (B) Molecular docking of STAT3 with chlorogenic acid (**29**). (C) Molecular docking of STAT3 with chicoric acid (**33**). (D) Molecular docking of STAT3 with luteolin-7-*O*-glucoside (**16**). (E) Molecular docking of NF-κB with chicoric acid (**33**). (F) Molecular docking of NF-κB with phillyrin (**41**).

Finally, we performed molecular docking analyses on the seven primary active compounds identified in the network pharmacology analysis, focusing on the key targets STAT3 and NF-κB. Docking results showed that conformations with high binding affinity (glide gscore < −6; Table 1) were STAT3 with forsythiaside A (**39**), chlorogenic acid (**29**), chicoric acid (**33**), and luteolin-7-*O*-glucoside (**16**), and NF-κB with chicoric acid (**33**) and phillyrin (**41**) (Fig. 8A–F). The above data show that the main active substances in FHF are forsythiaside A (**39**), chlorogenic acid (**29**), and chicoric acid (**33**).

**Table 1:** Score of molecular docking results.

Rank.	Compound	Target	glide_score
1	Forsythiaside A (**39**)	STAT3	−10.007
2	Chlorogenic acid (**29**)		−8.768
3	Chicoric acid (**33)**		−7.682
4	Luteolin-7-*O*-glucoside (**16**)		−7.667
5	Chicoric acid (**33**)	NF-κB	−7.605
6	Phillyrin (**41**)		−6.314

## 4 Discussion

Despite the presence of distinct clinical entities, persistent activation of the JAK/STAT pathway represents a common pathogenic characteristic across the disease spectrum. Mutations in JAK2, CALR, or MPL have been identified in the majority of patients [37]. Consequently, JAK inhibitors, such as ruxolitinib, have been approved for the clinical treatment of MPNs [38]. However, current clinical treatments have limitations, including adverse effects (such as anemia and thrombocytopenia), an inability to reverse bone marrow fibrosis, and failure to eliminate the malignant clone. More importantly, single-target therapies often struggle to address the complex inflammatory microenvironment in MPNs, which continuously promotes disease progression and leukemic transformation. Traditional Chinese medicine offers multi-target, multi-pathway regulatory advantages. In this study, we demonstrate for the first time across three MPN models that the classic Chinese herbal formula FHF significantly attenuates disease progression, effectively reduces myeloid cell expansion and splenomegaly, and even alleviates myelofibrosis and prolongs survival in the ET model. Whereas ruxolitinib primarily inhibits JAK1/2 signaling, FHF concurrently modulates both the STAT3 and NF-κB inflammatory pathways and activates p53/p21-dependent cellular senescence. This multi-targeted mechanism may mitigate the compensatory signaling responses frequently observed with single-pathway blockade. Furthermore, safety assessments conducted in this study revealed no hematological abnormalities or organ toxicity following 21 consecutive days of FHF administration, underscoring its favorable safety profile. Although a direct head-to-head comparison with ruxolitinib was not performed, the distinct mechanism of action and favorable tolerability observed here suggest that FHF could serve as an adjunct to conventional JAK inhibitor therapy. Such a strategy may allow for dose reduction of JAK inhibitors and amelioration of associated adverse effects while preserving or even potentiating therapeutic efficacy. Future investigations employing combination regimens are warranted to validate these potential synergistic effects. In our animal experiments, only a single dose level was used, and the dose-response relationship of FHF was not systematically evaluated. Future studies should include multiple dose groups combined with pharmacokinetic analysis to optimize the dosing regimen and define the therapeutic window of FHF. Moreover, colony-forming assays in this study were performed exclusively with CD34^+^ cells from JAK2^V617F^-positive MPN patients. Future clinical studies should encompass larger and more diverse patient cohorts, including those with CALR and MPL mutations, to more comprehensively evaluate the efficacy of FHF.

In this study, a total of 43 small-molecule components were identified by UHPLC-MS/MS, consisting primarily of alkaloids (9 compounds) and phenylpropanoids (9 compounds). Molecular docking with the key proteins STAT3 and NF-κB revealed that Chicoric acid exhibited high docking scores against both targets, suggesting that it may serve as a key pharmacologically active component in FHF, warranting further in-depth investigation. Notably, FHF also contains the animal medicine Scolopendra, which is rich in various bioactive peptides and is recognized as an important treasure trove for drug discovery [[39], [40], [41], [42], [43], [44]]. Recent studies have demonstrated that centipede-derived peptides possess anti-inflammatory, immunomodulatory, and anti-tumor activities, potentially acting through NF-κB and STAT3 signaling pathways. However, due to methodological limitations, the UHPLC-MS/MS analysis in this study focused primarily on small-molecule components and did not cover peptide components (peptides require different extraction and detection conditions). This represents an important gap in fully elucidating the active constituent profile of FHF. Future studies should focus on characterizing the peptide components of FHF and validating their potential effects, either individually or in synergy with small-molecule constituents, in the treatment of MPNs.

JAK/STAT signaling is interconnected with numerous core cancer signaling pathways, including but not limited to transcriptional control, apoptosis, cell cycle control, and the DNA damage response [45]. Evidence has demonstrated that the secretion of cytokines by both nonmalignant and malignant cells, driven by the JAK/STAT3 pathway, plays a pivotal role in MPN pathogenesis [24]. In addition, NF-κB signaling was identified as a significant pathway in both malignant and nonmalignant cells in MPNs [16], indicating a collaborative and co-regulatory relationship between NF-κB and STAT3 drivers in the inflammatory state of MPNs [16]. In this context, the present study demonstrates that FHF reduces NF-κB nuclear translocation, inhibits its translocation activity, and consequently lowers inflammatory factor expression in MPN cells. Importantly, the findings of this study demonstrate that the concurrent inhibition of the STAT3 and NF-κB signaling pathways by FHF results in a substantial enhancement of the therapeutic efficacy in MPNs. However, we have not yet validated the effects of FHF on the p53/p21, STAT3, or NF-κB pathways through loss-of-function experiments. Further in-depth studies are warranted using gene knockdown/silencing or pathway inhibitor approaches.

**Fig. 9:**
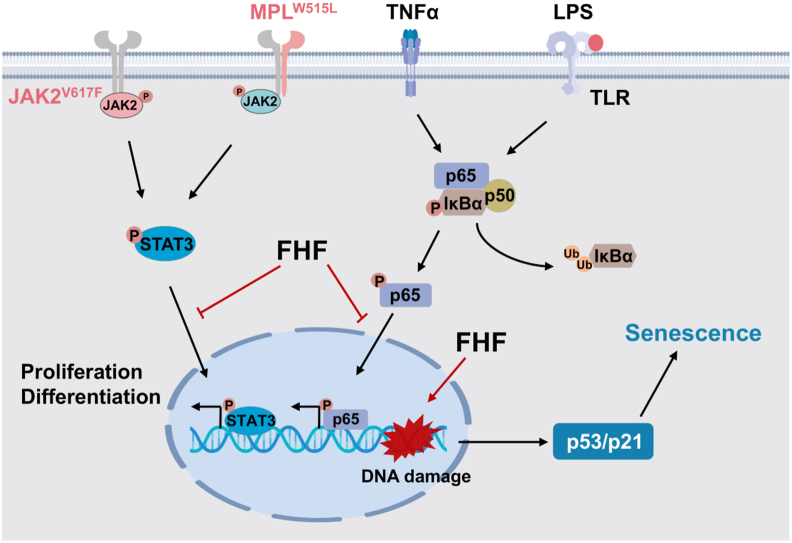
Schematic illustration of FHF treatment for MPNs. FHF treatment inhibits the JAK2/STAT3 and NF-κB signaling pathways. This suppresses the expression of pro-inflammatory cytokines and cell proliferation. On the other hand, FHF promotes cellular senescence by activating the p53/p21 signaling pathway.

In addition, network pharmacology and RNAseq enrichment analysis demonstrated that FHF modulates MPNs progression by acting on cell senescence, DNA replication, cell cycle regulation, and p53 signaling pathways. Multiple distinct factors drive senescence, with DDR representing a central factor in these mechanisms [46,47]. Sustained DDR triggers p53-dependent cell cycle arrest, and ultimately induces cellular senescence [48,49]. Furthermore, the activation of p53 signaling by inhibition of MDM2 has shown clinical promise in MPN treatment [50,51], while p53 deficiency has facilitated leukemic transformation in the JAK2^V617F^-driven MPN models [52]. Further experimental findings indicate that FHF inhibits MPN cell proliferation by inducing cell senescence. FHF significantly elevates the phosphorylation levels of H2AX, a DNA damage marker, and enhances p53 activation; subsequently, it upregulates the cyclin-dependent kinase (CDK) inhibitor p21 in MPN cells. While cellular senescence is characterized by the senescence-associated secretory phenotype (SASP), it is mainly driven by the NF-κB and cGAS-STING activation. However, sustained DDR signaling is imperative for SASP gene expression [47,53]. The data presented herein indicate that FHF activates DNA damage-induced cellular senescence while inhibiting the STAT3 and NF-κB signaling pathways (Fig. 9). This finding suggests that the inhibitory effect of FHF on STAT3 and NF-κB contributes to a reduction in cytokine release both within the disease microenvironment and during the cellular senescence process, ultimately achieving the goal of delaying the progression of MPNs. It should be noted that network pharmacology also predicted other potential pathways (e.g., Th17 cell differentiation, hematopoietic cell lineage). The present study focused primarily on senescence and inflammation, and these additional predicted pathways were not experimentally validated. Nevertheless, whether these pathways are associated with the efficacy of FHF in treating MPNs remains to be investigated in future studies.

## 5 Limitations

Limitations of this study: First, although our data demonstrate a significant correlation between FHF treatment and activation of the p53/p21 signaling pathway as well as inhibition of the STAT3/NF-κB pathways, we did not perform loss-of-function experiments. Second, the animal experiments employed only a single dose of FHF without a dose-gradient design or time-response evaluation, and pharmacokinetic monitoring of the major active components was not performed. Third, patient sample experiments were confined to colony-forming assays using CD34^+^ cells from a limited number of JAK2^V617F^-positive MPN patients, without the inclusion of other mutation subtypes. Fourth, although network pharmacology predicted other potential pathways (e.g., Th17 cell differentiation, hematopoietic cell lineage), experimental validation was focused solely on the senescence- and inflammation-related pathways p53/p21, STAT3, and NF-κB. Future studies should employ genetic loss-of-function and pharmacological intervention approaches to establish the causal roles of these pathways, conduct dose-response and pharmacokinetic analyses, and validate the efficacy of FHF in larger patient cohorts encompassing different MPN subtypes.

## 6 Conclusion

Our study is the first to demonstrate the therapeutic efficacy of FHF in the context of MPNs. This efficacy is underscored by its capability to modulates STAT3 and NF-κB signaling, while concurrently inducing cellular senescence through the DNA damage response pathway, specifically via the p53/p21 signaling cascade (Fig. 9).

## CRediT authorship contribution statement

**Mingjie Liu:** Writing – original draft, Methodology, Investigation, Data curation. **Yanxia Li:** Visualization, Validation. **Chengxue Qin:** Resources, Investigation. **Lingling Wang:** Software, Investigation. **Meiqi Guo:** Visualization, Validation. **Yi Wang:** Methodology, Formal analysis. **Qian Zhou:** Software, Methodology, Investigation. **Zhida Shi:** Project administration, Conceptualization. **Weimin Hao:** Resources, Project administration. **Yuan Li:** Supervision, Project administration. **Baobing Zhao:** Writing – review & editing, Resources, Project administration, Funding acquisition.

## Ethics approval and consent to participate

The design and performing of animal experiments were approved by the Institutional Animal Care and Use Committees at Shandong University. APPROVAL NUMBER: KYLL-2023(ZM)-576.

## Declaration of generative AI in scientific writing

No generative AI tools have been used throughout the entire writing process of this manuscript.

## Funding

We thank the Translational Medicine Core Facility of Shandong University for the availability of consultation and instruments that supported this work. This work was supported by grants from the 10.13039/501100012166National Key Research and Development Program of China (2024YFC2510500), 10.13039/501100001809National Natural Science Foundation of China (81874294, 82200989), 10.13039/501100007129Natural Science Foundation of Shandong Province (ZR2024MH065) and the key Program of Innovation Improvement of Small and Medium-sized Enterprises (2023TSGC0717) of Shandong Province in China.

## Declaration of competing interests

The authors declare that they have no known competing financial interests or personal relationships that could have appeared to influence the work reported in this paper.

## Data Availability

The datasets used and/or analyzed during the current study are available from the corresponding author on reasonable request. RNA-seq data that support the findings of this study are openly available in [https://www.ncbi.nlm.nih.gov/geo/query/acc.cgi?acc=GSE252715], reference number [GSE252715].
