# Supplementary material for: Fufang Huangbo Formula mitigates myeloproliferative neoplasms by activating p53/p21 signaling axis and inhibiting STAT3 and NF-κB signaling pathways
Source: Pharm Sci Adv. 2026 Apr 16;4:100121. doi: 10.1016/j.pscia.2026.100121 (PMC13129468; doi:10.1016/j.pscia.2026.100121)
Supplement: Multimedia component 1 [file mmc1.docx]

**Fufang Huangbo Formula mitigates myeloproliferative neoplasms by activating p53/p21 signaling axis and inhibiting STAT3 and NF-κB signaling pathways**

Mingjie Liu^1-4^, Yanxia Li^1-4^, Chengxue Qin^1-4^, Lingling Wang^1-4^, Meiqi Guo^1-4^, Yi Wang^1-4^, Qian Zhou^1-4^, Zhida Shi^5*^, Weimin Hao^6*^, Yuan Li^1-4*^, Baobing Zhao^1-4*^

**Affiliations**

^1^ State Key Laboratory of Discovery and Utilization of Functional Components in Traditional Chinese Medicine, Shandong University, Jinan, Shandong, 250012, China

^2^ Key Lab of Chemical Biology (MOE), Shandong University, Jinan, Shandong, 250012, China

^3^ NMPA Key Laboratory for Technology Research and Evaluation of Drug Products, Shandong University, Jinan, Shandong 250012, China

^4^ Department of Pharmacology, School of Pharmaceutical Sciences, Cheeloo College of Medicine, Shandong University, Jinan, Shandong, 250012, China

^5^ Department of Spine Surgery, Heze municipal Hospital, 2888 Caozhou Road, Heze, Shandong, 274031, China.

^6^ Research Center for Traditional Chinese Medicine and Clinical Pharmacy, Shandong Provincial Maternal and Child Health Care Hospital, Jinan, Shandong, 250012, China

**Correspondence**

^*^Department of Pharmacology, School of Pharmaceutical Sciences, Shandong University, 44 W Wenhua Road, Jinan, Shandong, China.

E-mail address: [baobingzh@sdu.edu.cn](mailto:baobingzh@sdu.edu.cn) (Baobing Zhao), [liyuan23@sdu.edu.cn](mailto:liyuan23@sdu.edu.cn) (Yuan Li); TEL/FAX: +86-531-88382176

^**^Department of Spine Surgery, Heze municipal Hospital, 2888 Caozhou Road, Heze, Shandong, 274031, China.

E-mail address: haoweimin830125@163.com

^***^Research Center for Traditional Chinese Medicine and Clinical Pharmacy, Shandong Provincial Maternal and Child Health Care Hospital, 12675, Jing Shisan East Road, Jinan, Shandong, China.

E-mail address: shizhida1@126.com

**Supplementary Information**


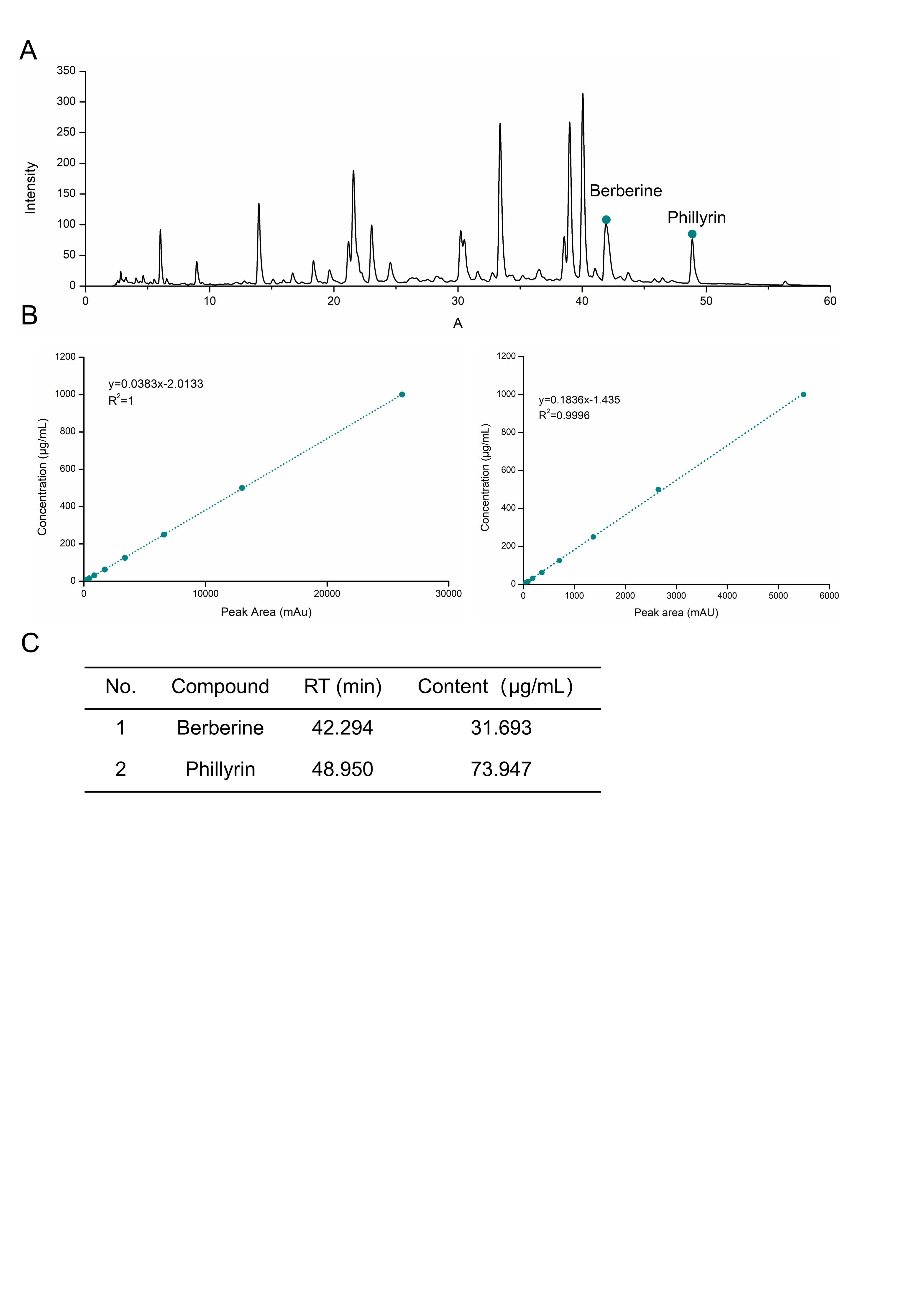


**Fig. S1.** Quantitative analysis of FHF. (A) The HPLC chromatograms of FHF. (B-C) Standard curves and content of berberine and phillyrin. The analysis of FHF content was performed on an Agilent 1260 system equipped with degasser, auto sampler, and diode array detector. The separation was performed with a EC-C18 HPLC column (4.6*150 mm, 5 μm), and eluted with a MeOH-H_2_O elution. The detective wavelength was set on 278 nm.

**
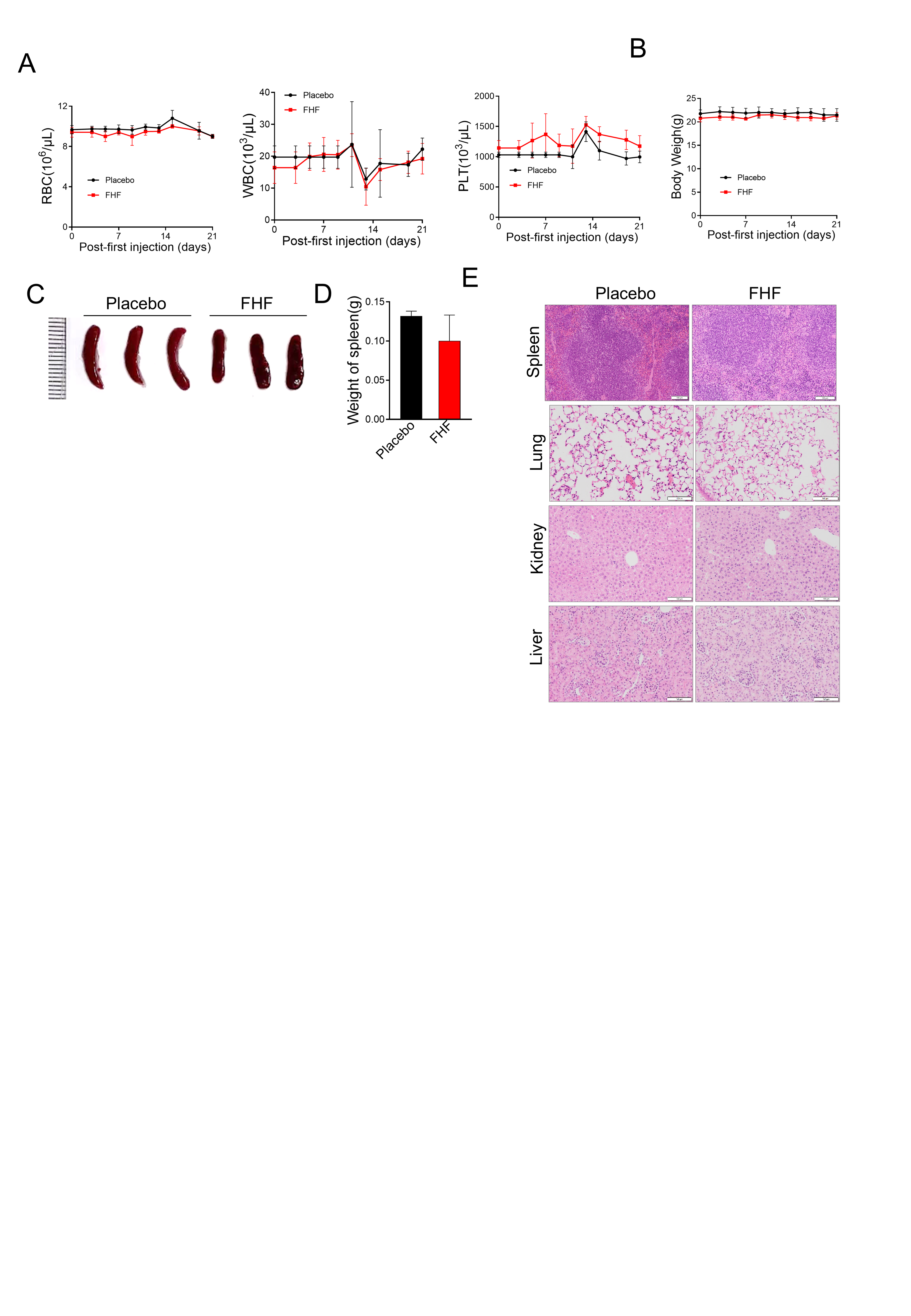
**

**Fig. S2.** FHF did not affect the hematopoietic function of normal mouse. (A) RBC, WBC and PLT in peripheral blood from indicated mice after treatment of FHF or placebo. (B) The body weight of the mice from indicated group mice. (C) Representative spleen from indicated group mic after 21 days of treatment. (D) Statistical analysis of spleen weights in C. (E) Representative H&E staining of spleen, lung, kidney and liver from indicated mice. RBC: red blood cell; WBC: white blood cell; PLT: platelet;


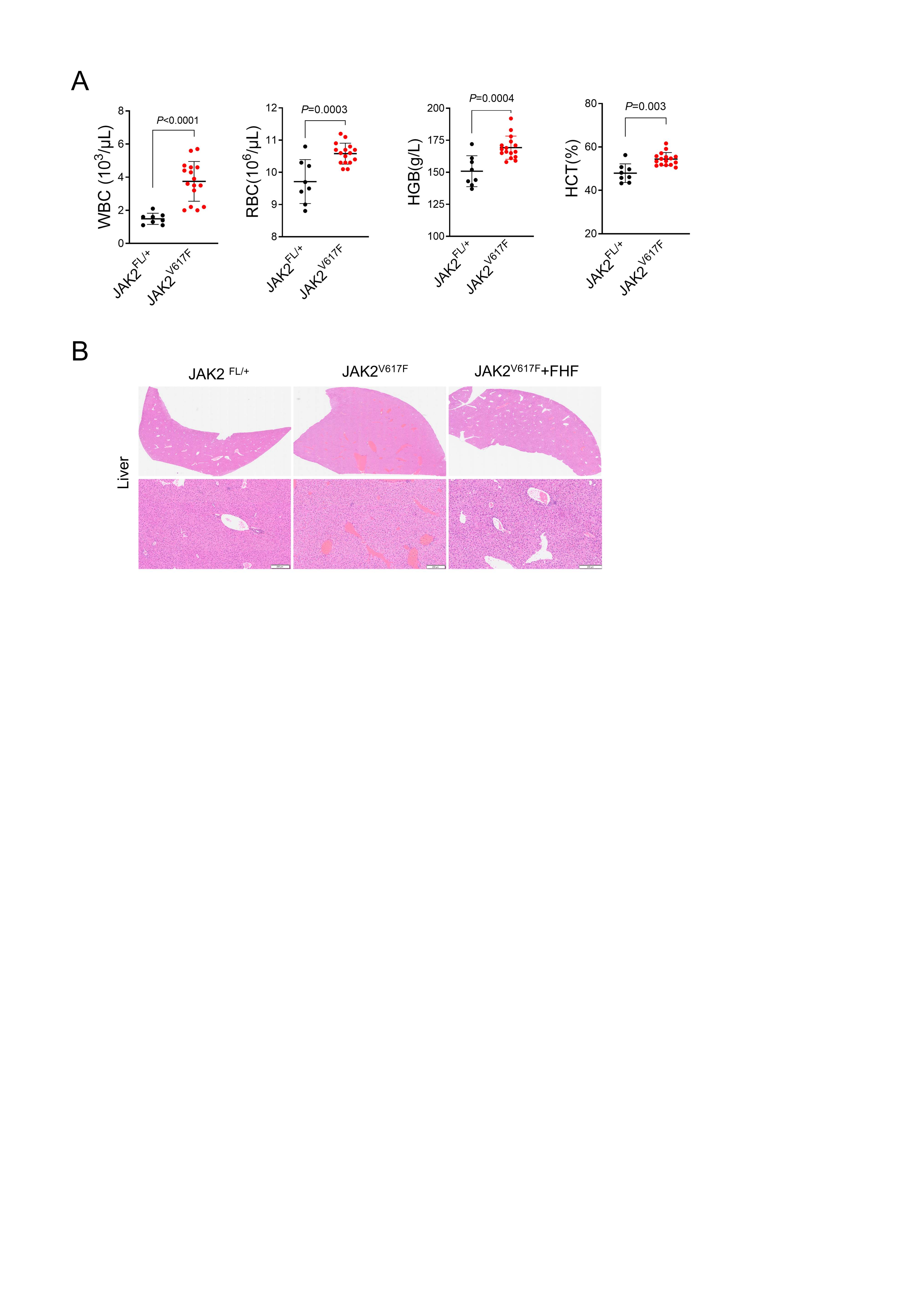


**Fig. S3.** Effect of FHF administration on JAK2^V617F^-induced MPN mice. (A) WBC, RBC, HGB and HCT in peripheral blood from indicated mice after two weeks of bone marrow transplantation. Each dot represents one mouse. (B) Representative H&E staining of liver from indicated mice. WBC: white blood cell; RBC: red blood cell; HGB: Hemoglobin; HCT: Hematocrit.

**
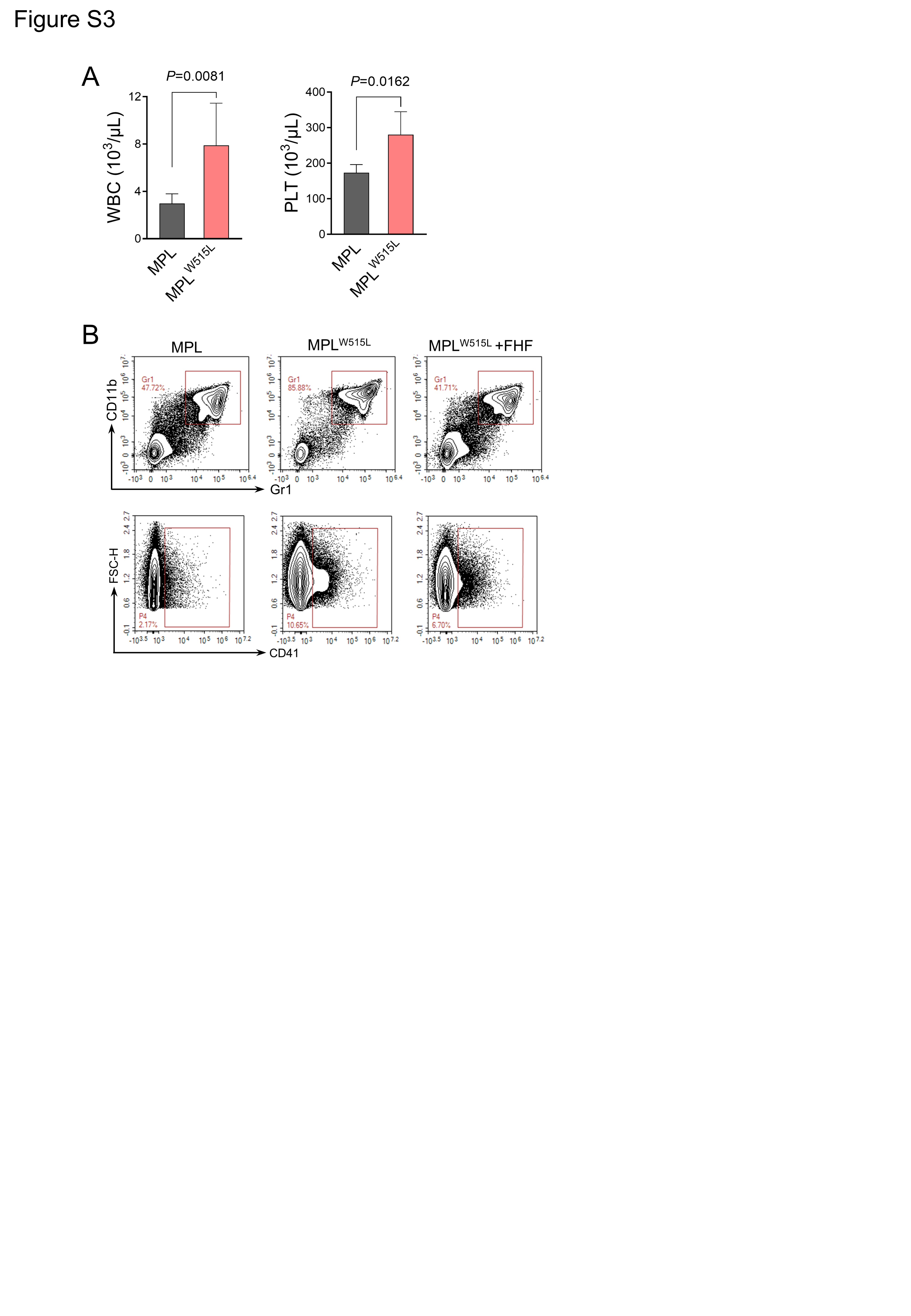
**

**Fig. S4** Effect of FHF administration on MPL^W515L^-driven mouse model. (A) WBC and PLT in peripheral blood from indicated mice after two weeks of bone marrow transplantation. N=6 mice per group*.* (B) Representative flow cytometric analysis of myeloid cells (Gr1^+^CD11b^+^) and megakaryocytes (CD41^+^) in bone marrow from indicated mice. WBC: white blood cell; PLT: platelet.

**
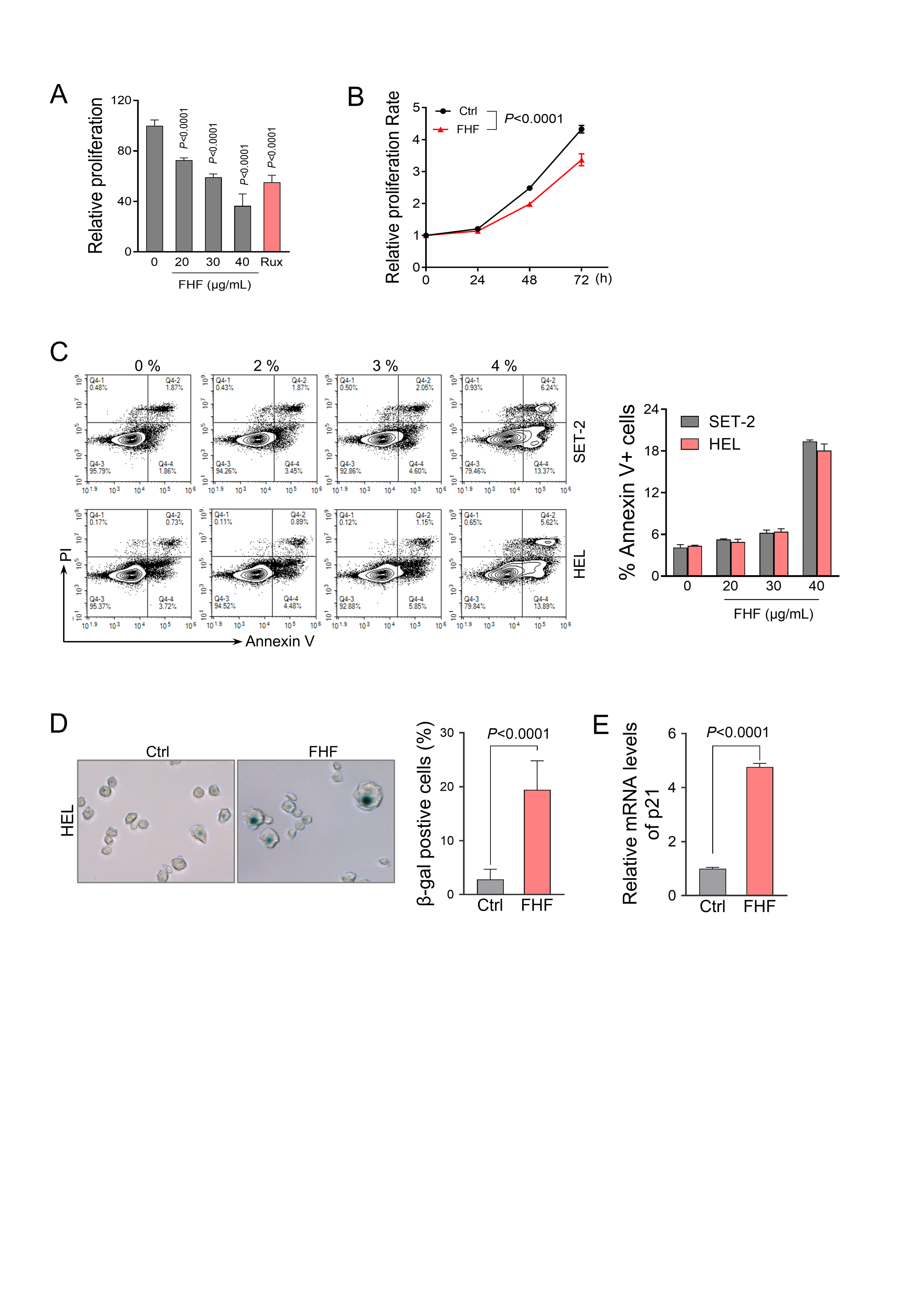
**

**Fig. S5** Effect of FHF on the proliferation of JAK2^V617F^ expressing cells. (A) Statistical analysis of cell counts in HEL cells with FHF or Rux. (B) Statistical analysis of cell proliferation in HEL cells with FHF or Rux. Ctrl represents vehicle control. *P* value was determined by two-way ANOVA. (C) Flow cytometric analysis of cell apoptosis in SET-2 and HEL cell with FHF treatment for 24 h. (D) Representative images of SA-β-gal staining in HEL cells with FHF in the presence of Dox (0.3 μM) for 24 h. Quantification of SA-β-gal positive cells was shown. (E) Quantitative PCR analysis of p21 mRNA levels in HEL cells with FHF treatment for 12 h. SA-β-gal: senescence-associated-β-galactosidase; Rux: ruxolitinib; Dox: doxorubicin.

**
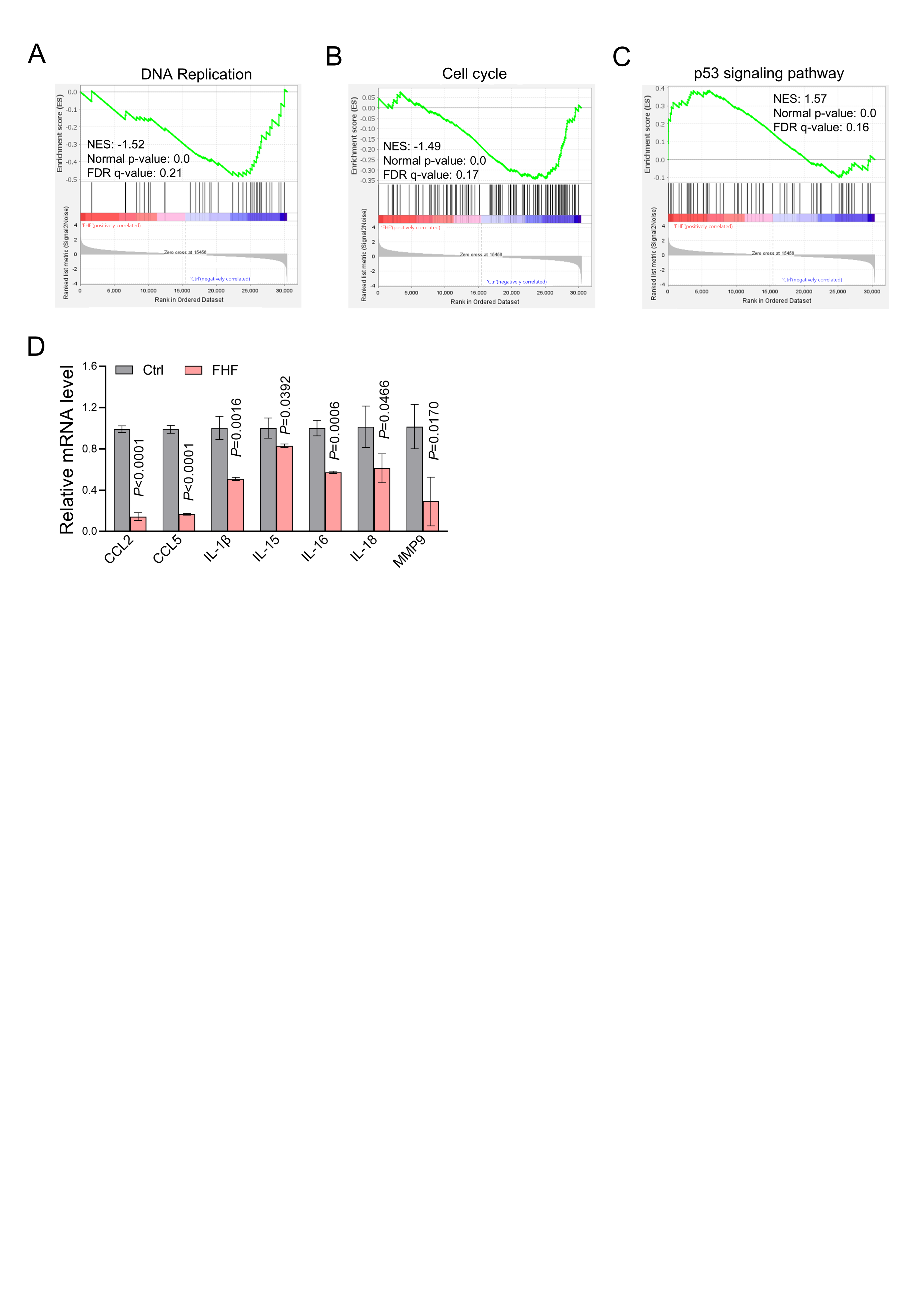
**

**Fig. S6** RNAseq analysis of FHF-treated JAK2^V617F^ expressing cells. (A-C) Gene Set Enrichment Analysis of DNA replication, cell cycle and p53 signaling pathway in SET-2 cells treated by FHF. (D) Quantitative PCR analysis of indicated genes in SET-2 cells with FHF treatment for 8 h.

**Supplemental Tables**

**Table S1.** The chemical components of FHF identified by UHPLC-MS/MS.

| **NO.** | **RT [min]** | **Identification** | **Molecular Formula** | **Theoretical mass *m/z*** | **Experimental mass *m/z*** | **Adducts** | **Category** |
| --- | --- | --- | --- | --- | --- | --- | --- |
| 1 | 1.01 | L-Proline | C_5_ H_9_ N O_2_ | 116.07061 | 116.07082 | [M+H]^+^ | Alkaloids |
| 2 | 1.38 | L-Pyroglutamic acid | C_5_ H_7_ N O_3_ | 130.04987 | 130.04988 | [M+H]^+^ | Alkaloids |
| 3 | 1.58 | Homoanatoxin A | C_11_ H_17_ N O | 180.13829 | 180.13815 | [M+H]^+^ | Alkaloids |
| 4 | 1.75 | Leucine | C_6_ H_13_ N O_2_ | 132.10191 | 132.10188 | [M+H]^+^ | Alkaloids |
| 5 | 2.03 | 2-Methoxybenzaldehyde | C_8_ H_8_ O_2_ | 137.05971 | 137.0596 | [M+H]^+^ | Others |
| 6 | 2.65 | Rengyoside A | C_14_ H_26_ O_8_ | 345.15199 | 345.15137 | [M+Na]^+^ | Others |
| 7 | 2.81 | Phenylacetylene | C_8_ H_6_ | 120.08077 | 120.08093 | [M+NH_4_]^+^ | Others |
| 8 | 7.47 | Methyl atratate | C_10_ H_12_ O_4_ | 197.0806 | 197.08084 | [M+H]^+^ | Phenolic acids |
| 9 | 7.99 | Phellodendrine | C_20_ H_24_ N O_4_ | 343.1773 | 343.17886 | [M+H]^+^ | Alkaloids |
| 10 | 11.67 | Forsythoside E | C_20_ H_30_ O_12_ | 485.16295 | 485.16223 | [M+Na]+ | Phenolic acids |
| 11 | 13.13 | 3-ethyl-7hydroxyphthalide | C_10_ H_10_ O_3_ | 179.07027 | 179.07021 | [M+H]^+^ | Phenolic acids |
| 12 | 14.08 | Isocorypalmine | C_20_ H_23_ N O_4_ | 342.16999 | 342.16956 | [M+H]^+^ | Alkaloids |
| 13 | 15.52 | Secologanic acid | C_10_ H_12_ O_5_ | 213.07575 | 213.07539 | [M+H]^+^ | Terpenoids |
| 14 | 16.19 | Vanillic acid | C_8_ H_8_ O_4_ | 151.03898 | 151.03879 | [M+H-H_2_O]^+^ | Phenolic acids |
| 15 | 25.85 | *p*-Coumaric acid | C_9_ H_8_ O_2_ | 165.05462 | 165.05452 | [M+H]^+^ | Phenolic acids |
| 16 | 33.85 | Luteolin-7-o-glucoside | C_21_ H_20_ O_11_ | 449.10784 | 449.10712 | [M+H]^+^ | Flavonoids |
| 17 | 35.14 | Forsythialan B | C_21_ H_24_ O_7_ | 389.15948 | 389.15889 | [M+H]^+^ | Phenolic acids |
| 18 | 36.13 | Daldiniside B | C_15_ H_16_ O_8_ | 325.0918 | 325.09113 | [M+H]^+^ | Phenylpropanoids |
| 19 | 41.84 | Safynol | C_13_ H_12_ O_2_ | 201.09101 | 201.09058 | [M+H]^+^ | Alkynes |
| 20 | 48.16 | Clorius | C_8_ H_8_ O_2_ | 137.05971 | 137.05954 | [M+H]^+^ | Others |
| 21 | 56.05 | 6-acetyl-4,4,7-trimethylbicyclo[4.1.0]heptan-2-one | C_12_ H_18_ O_2_ | 195.13796 | 195.13762 | [M+H]^+^ | Terpenoids |
| 22 | 58.53 | Phthalic anhydride | C_8_ H_4_ O_3_ | 149.02332 | 149.02318 | [M+H]^+^ | Phenolic acids |
| 23 | 0.99 | [(Carbamoylamino)methyl]carbamate | C_3_ H_6_ N_3_ O_3_ | 191.05531 | 191.05527 | [M-H+HAc]^-^ | Alkaloids |
| 24 | 2.05 | Vanilloloside | C_14_ H_20_ O_8_ | 315.10854 | 315.18024 | [M-H]^-^ | Phenolic acids |
| 25 | 2.5 | Forsythidmethylester | C_11_ H_14_ O_6_ | 301.09289 | 301.09326 | [M-H+HAc]^-^ | Terpenoids |
| 26 | 2.71 | Rengyolone | C_8_ H_14_ O_4_ | 173.08193 | 173.08136 | [M-H]^-^ | Others |
| 27 | 3.00 | L-Phenylalanine | C_9_ H_11_ N O_2_ | 164.0717 | 164.07103 | [M-H]^-^ | Alkaloids |
| 28 | 7.58 | Berberine | C_20_ H_18_ N O_4_ | 335.11575 | 335.11469 | [M-H]^-^ | Alkaloids |
| 29 | 11.52 | Chlorogenic acid | C_16_ H_18_ O_9_ | 353.0878 | 353.08795 | [M-H]^-^ | Phenylpropanoids |
| 30 | 15.62 | Secologanate | C_17_ H_24_ O_9_ | 373.11402 | 373.11407 | [M-H]^-^ | Others |
| 31 | 16.17 | Forsythide | C_16_ H_22_ O_11_ | 389.10893 | 389.10913 | [M-H]^-^ | Terpenoids |
| 32 | 23.38 | [(2R,3S,4S,5R,6R)-3,4,5-trihydroxy-6-[2-(1-hydroxy-4-oxo-1-cyclohexa-2,5-dienyl)ethoxy]oxan-2-yl]methyl 2-(4-hydroxyphenyl)acetate | C_22_ H_26_ O_10_ | 449.14532 | 449.14584 | [M-H]^-^ | Phenolic acids |
| 33 | 23.47 | Chicoric acid | C_22_ H_18_ O_12_ | 473.07255 | 473.07312 | [M-H]^-^ | Phenylpropanoids |
| 34 | 27.6 | Phellavin | C_26_ H_32_ O_12_ | 535.1821 | 535.18262 | [M-H]^-^ | Flavonoids |
| 35 | 28.69 | Forsythoside C | C_29_ H_36_ O_16_ | 639.19439 | 639.19464 | [M-H]^-^ | Phenylpropanoids |
| 36 | 33.83 | (+)-epipinoresinol-4'-O-D-glucoside | C_26_ H_32_ O_11_ | 519.18718 | 519.18756 | [M-H]^-^ | Phenylpropanoids |
| 37 | 34.65 | Rutin | C_27_ H_30_ O_16_ | 609.1461 | 609.14691 | [M-H]^-^ | Flavonoids |
| 38 | 35.34 | Isochlorogenic acid | C_25_ H_24_ O_12_ | 515.1195 | 515.11938 | [M-H]^-^ | Phenylpropanoids |
| 39 | 36.15 | Forsythiaside A | C_29_ H_36_ O_15_ | 623.19814 | 623.19817 | [M-H]^-^ | Phenylpropanoids |
| 40 | 39.64 | Ethyl caffeate | C_11_ H_12_ O_4_ | 207.06628 | 207.06596 | [M-H]^-^ | Phenylpropanoids |
| 41 | 41.86 | Phillyrin | C_27_ H_34_ O_11_ | 533.20283 | 533.20361 | [M-H]^-^ | Phenylpropanoids |
| 42 | 54.73 | Malyngic acid | C_18_ H_32_ O_5_ | 327.21769 | 327.21793 | [M-H]^-^ | Others |
| 43 | 56.03 | Penicitide B | C_18_ H_34_ O_5_ | 329.23334 | 329.23352 | [M-H]^-^ | Others |

**Table S2.** The chemical components ID of FHF in the treatment of MPN.

| **ID** | **Chemical Component** | **Molecular Formula** | **Category** |
| --- | --- | --- | --- |
| T.1 | L-Proline | C_5_ H_9_ N O_2_ | Alkaloids |
| T.2 | L-Pyroglutamic acid | C_5_ H_7_ N O_3_ | Alkaloids |
| T.3 | Homoanatoxin A | C_11_ H_17_ N O | Alkaloids |
| T.4 | Leucine | C_6_ H_13_ N O_2_ | Alkaloids |
| T.5 | 2-Methoxybenzaldehyde | C_8_ H_8_ O_2_ | Others |
| T.6 | Rengyoside A | C_14_ H_26_ O_8_ | Others |
| T.7 | Phenylacetylene | C_8_ H_6_ | Others |
| T.8 | Methyl atratate | C_10_ H_12_ O_4_ | Phenolic acids |
| T.9 | Phellodendrine | C_20_ H_24_ N O_4_ | Alkaloids |
| T.10 | Forsythoside E | C_20_ H_30_ O_12_ | Phenolic acids |
| T.11 | 3-ethyl-7hydroxyphthalide | C_10_ H_10_ O_3_ | Phenolic acids |
| T.12 | Isocorypalmine | C_20_ H_23_ N O_4_ | Alkaloids |
| T.13 | Secologanic acid | C_10_ H_12_ O_5_ | Terpenoids |
| T.14 | Vanillic acid | C_8_ H_8_ O_4_ | Phenolic acids |
| T.15 | *p*-Coumaric acid | C_9_ H_8_ O_2_ | Phenolic acids |
| T.16 | Luteolin-7-o-glucoside | C_21_ H_20_ O_11_ | Flavonoids |
| T.17 | Forsythialan B | C_21_ H_24_ O_7_ | Phenolic acids |
| T.18 | Daldiniside B | C_15_ H_16_ O_8_ | Phenylpropanoids |
| T.19 | Safynol | C_13_ H_12_ O_2_ | Alkynes |
| T.20 | Clorius | C_8_ H_8_ O_2_ | Others |
| T.21 | 6-acetyl-4,4,7-trimethylbicyclo[4.1.0]heptan-2-one | C_12_ H_18_ O_2_ | Terpenoids |
| T.22 | Phthalic anhydride | C_8_ H_4_ O_3_ | Phenolic acids |
| T.23 | [(Carbamoylamino)methyl]carbamate | C_3_ H_6_ N_3_ O_3_ | Alkaloids |
| T.24 | Vanilloloside | C_14_ H_20_ O_8_ | Phenolic acids |
| T.25 | Forsythidmethylester | C_11_ H_14_ O_6_ | Terpenoids |
| T.26 | Rengyolone | C_8_ H_14_ O_4_ | Others |
| T.27 | L-Phenylalanine | C_9_ H_11_ N O_2_ | Alkaloids |
| T.28 | Berberine | C_20_ H_18_ N O_4_ | Alkaloids |
| T.29 | Chlorogenic acid | C_16_ H_18_ O_9_ | Phenylpropanoids |
| T.30 | Secologanate | C_17_ H_24_ O_9_ | Others |
| T.31 | Forsythide | C_16_ H_22_ O_11_ | Terpenoids |
| T.32 | [(2R,3S,4S,5R,6R)-3,4,5-trihydroxy-6-[2-(1-hydroxy-4-oxo-1-cyclohexa-2,5-dienyl)ethoxy]oxan-2-yl]methyl 2-(4-hydroxyphenyl)acetate | C_22_ H_26_ O_10_ | Phenolic acids |
| T.33 | Chicoric acid | C_22_ H_18_ O_12_ | Phenylpropanoids |
| T.34 | Phellavin | C_26_ H_32_ O_12_ | Flavonoids |
| T.35 | Forsythoside C | C_29_ H_36_ O_16_ | Phenylpropanoids |
| T.36 | (+)-epipinoresinol-4'-O-D-glucoside | C_26_ H_32_ O_11_ | Phenylpropanoids |
| T.37 | Rutin | C_27_ H_30_ O_16_ | Flavonoids |
| T.38 | Isochlorogenic acid | C_25_ H_24_ O_12_ | Phenylpropanoids |
| T.39 | Forsythiaside A | C_29_ H_36_ O_15_ | Phenylpropanoids |
| T.40 | Ethyl caffeate | C_11_ H_12_ O_4_ | Phenylpropanoids |
| T.41 | Phillyrin | C_27_ H_34_ O_11_ | Phenylpropanoids |
| T.42 | Malyngic acid | C_18_ H_32_ O_5_ | Others |
| T.43 | Penicitide B | C_18_ H_34_ O_5_ | Others |

**Table S3**. GSEA enrichment analysis of the differentially expressed genes from RNA sequencing of SET-2 cells treated FHF.

| **No** | **Genesets** | **SIZE** | **ES** | **NES** | **NOM p-val** | **FDR q-val** | **FWER p-val** |
| --- | --- | --- | --- | --- | --- | --- | --- |
| 1 | KEGG_CYTOKINE_CYTOKINE_RECEPTOR_INTERACTION | 196 | 0.45 | 1.74 | 0 | 0.087 | 0.038 |
| 2 | KEGG_ERBB_SIGNALING_PATHWAY | 82 | 0.54 | 1.67 | 0 | 0.188 | 0.264 |
| 3 | KEGG_JAK_STAT_SIGNALING_PATHWAY | 117 | 0.4 | 1.64 | 0 | 0.142 | 0.264 |
| 4 | KEGG_BLADDER_CANCER | 39 | 0.6 | 1.62 | 0 | 0.159 | 0.318 |
| 5 | KEGG_STEROID_HORMONE_BIOSYNTHESIS | 35 | 0.46 | 1.61 | 0 | 0.179 | 0.318 |
| 6 | KEGG_METABOLISM_OF_XENOBIOTICS_BY_CYTOCHROME_P450 | 47 | 0.49 | 1.6 | 0 | 0.166 | 0.367 |
| 7 | KEGG_ADHERENS_JUNCTION | 71 | 0.46 | 1.59 | 0 | 0.149 | 0.367 |
| 8 | KEGG_REGULATION_OF_AUTOPHAGY | 18 | 0.51 | 1.59 | 0.084 | 0.151 | 0.421 |
| 9 | KEGG_CHEMOKINE_SIGNALING_PATHWAY | 165 | 0.37 | 1.58 | 0 | 0.164 | 0.502 |
| 10 | KEGG_TYPE_II_DIABETES_MELLITUS | 43 | 0.49 | 1.57 | 0 | 0.159 | 0.502 |
| 11 | KEGG_P53_SIGNALING_PATHWAY | 63 | 0.39 | 1.57 | 0 | 0.162 | 0.502 |
| 12 | KEGG_EPITHELIAL_CELL_SIGNALING_IN_HELICOBACTER_PYLORI_INFECTION | 64 | 0.56 | 1.57 | 0 | 0.153 | 0.502 |
| 13 | KEGG_GNRH_SIGNALING_PATHWAY | 90 | 0.44 | 1.57 | 0 | 0.145 | 0.502 |
| 14 | KEGG_REGULATION_OF_ACTIN_CYTOSKELETON | 193 | 0.38 | 1.55 | 0 | 0.165 | 0.548 |
| 15 | KEGG_GLIOMA | 60 | 0.37 | 1.55 | 0 | 0.164 | 0.548 |
| 16 | KEGG_VASOPRESSIN_REGULATED_WATER_REABSORPTION | 42 | 0.37 | 1.53 | 0 | 0.187 | 0.591 |
| 17 | KEGG_VIBRIO_CHOLERAE_INFECTION | 51 | 0.49 | 1.53 | 0 | 0.179 | 0.591 |
| 18 | KEGG_FOCAL_ADHESION | 189 | 0.4 | 1.53 | 0 | 0.174 | 0.591 |
| 19 | KEGG_FC_GAMMA_R_MEDIATED_PHAGOCYTOSIS | 93 | 0.4 | 1.52 | 0 | 0.167 | 0.591 |
| 20 | KEGG_T_CELL_RECEPTOR_SIGNALING_PATHWAY | 98 | 0.38 | 1.52 | 0 | 0.177 | 0.639 |
| 21 | KEGG_MAPK_SIGNALING_PATHWAY | 241 | 0.45 | 1.51 | 0 | 0.177 | 0.697 |
| 22 | KEGG_TOLL_LIKE_RECEPTOR_SIGNALING_PATHWAY | 87 | 0.47 | 1.51 | 0 | 0.171 | 0.697 |
| 23 | KEGG_PROSTATE_CANCER | 84 | 0.39 | 1.5 | 0 | 0.171 | 0.697 |
| 24 | KEGG_NOD_LIKE_RECEPTOR_SIGNALING_PATHWAY | 56 | 0.43 | 1.49 | 0 | 0.177 | 0.755 |
| 25 | KEGG_PATHWAYS_IN_CANCER | 299 | 0.37 | 1.48 | 0 | 0.182 | 0.755 |
| 26 | KEGG_ECM_RECEPTOR_INTERACTION | 80 | 0.47 | 1.48 | 0 | 0.177 | 0.755 |
| 27 | KEGG_PHOSPHATIDYLINOSITOL_SIGNALING_SYSTEM | 71 | 0.36 | 1.48 | 0 | 0.176 | 0.755 |
| 28 | KEGG_NEUROTROPHIN_SIGNALING_PATHWAY | 120 | 0.42 | 1.47 | 0 | 0.172 | 0.755 |
| 29 | KEGG_ENDOCYTOSIS | 175 | 0.42 | 1.47 | 0 | 0.177 | 0.817 |
| 30 | KEGG_VEGF_SIGNALING_PATHWAY | 70 | 0.51 | 1.46 | 0 | 0.182 | 0.817 |
| 31 | KEGG_CHRONIC_MYELOID_LEUKEMIA | 70 | 0.39 | 1.44 | 0 | 0.191 | 0.868 |
| 32 | KEGG_SNARE_INTERACTIONS_IN_VESICULAR_TRANSPORT | 37 | 0.32 | 1.44 | 0 | 0.19 | 0.868 |
| 33 | KEGG_RIBOFLAVIN_METABOLISM | 15 | 0.51 | 1.43 | 0 | 0.191 | 0.868 |
| 34 | KEGG_WNT_SIGNALING_PATHWAY | 136 | 0.36 | 1.43 | 0 | 0.187 | 0.868 |
| 35 | KEGG_PROTEASOME | 44 | 0.51 | 1.43 | 0.082 | 0.191 | 0.868 |
| 36 | KEGG_RIG_I_LIKE_RECEPTOR_SIGNALING_PATHWAY | 52 | 0.44 | 1.42 | 0 | 0.191 | 0.868 |
| 37 | KEGG_AMINO_SUGAR_AND_NUCLEOTIDE_SUGAR_METABOLISM | 41 | 0.3 | 1.4 | 0 | 0.209 | 0.936 |
| 38 | KEGG_AXON_GUIDANCE | 114 | 0.41 | 1.4 | 0 | 0.205 | 0.936 |
| 39 | KEGG_THYROID_CANCER | 27 | 0.34 | 1.39 | 0 | 0.218 | 0.936 |
| 40 | KEGG_N_GLYCAN_BIOSYNTHESIS | 46 | 0.31 | 1.38 | 0 | 0.214 | 0.936 |
| 41 | KEGG_MELANOMA | 60 | 0.37 | 1.38 | 0 | 0.217 | 0.936 |
| 42 | KEGG_FC_EPSILON_RI_SIGNALING_PATHWAY | 71 | 0.37 | 1.37 | 0 | 0.222 | 0.936 |
| 43 | KEGG_TYROSINE_METABOLISM | 36 | 0.42 | 1.36 | 0 | 0.223 | 0.936 |
| 44 | KEGG_MATURITY_ONSET_DIABETES_OF_THE_YOUNG | 16 | 0.52 | 1.36 | 0.117 | 0.221 | 1 |
| 45 | KEGG_OTHER_GLYCAN_DEGRADATION | 15 | 0.58 | 1.35 | 0 | 0.22 | 1 |
| 46 | KEGG_TGF_BETA_SIGNALING_PATHWAY | 74 | 0.36 | 1.35 | 0.113 | 0.216 | 1 |
| 47 | KEGG_B_CELL_RECEPTOR_SIGNALING_PATHWAY | 74 | 0.37 | 1.35 | 0 | 0.218 | 1 |
| 48 | KEGG_LEUKOCYTE_TRANSENDOTHELIAL_MIGRATION | 104 | 0.3 | 1.35 | 0 | 0.221 | 1 |
| 49 | KEGG_ADIPOCYTOKINE_SIGNALING_PATHWAY | 57 | 0.4 | 1.34 | 0.195 | 0.224 | 1 |
| 50 | KEGG_NATURAL_KILLER_CELL_MEDIATED_CYTOTOXICITY | 108 | 0.3 | 1.34 | 0 | 0.221 | 1 |
| 51 | KEGG_INSULIN_SIGNALING_PATHWAY | 123 | 0.34 | 1.33 | 0 | 0.221 | 1 |
| 52 | KEGG_INOSITOL_PHOSPHATE_METABOLISM | 52 | 0.36 | 1.33 | 0 | 0.218 | 1 |
| 53 | KEGG_GAP_JUNCTION | 81 | 0.36 | 1.33 | 0 | 0.217 | 1 |
| 54 | KEGG_COLORECTAL_CANCER | 61 | 0.35 | 1.33 | 0 | 0.214 | 1 |
| 55 | KEGG_PHENYLALANINE_METABOLISM | 17 | 0.53 | 1.33 | 0.118 | 0.211 | 1 |
| 56 | KEGG_SPHINGOLIPID_METABOLISM | 36 | 0.44 | 1.32 | 0 | 0.212 | 1 |
| 57 | KEGG_MELANOGENESIS | 91 | 0.34 | 1.31 | 0 | 0.226 | 1 |
| 58 | KEGG_ENDOMETRIAL_CANCER | 50 | 0.39 | 1.29 | 0.175 | 0.246 | 1 |
| 59 | KEGG_PANCREATIC_CANCER | 66 | 0.36 | 1.29 | 0 | 0.246 | 1 |
| 60 | KEGG_HEMATOPOIETIC_CELL_LINEAGE | 73 | 0.34 | 1.29 | 0 | 0.246 | 1 |
| 61 | KEGG_GLYCEROPHOSPHOLIPID_METABOLISM | 71 | 0.36 | 1.29 | 0 | 0.243 | 1 |
| 62 | KEGG_ABC_TRANSPORTERS | 41 | 0.37 | 1.29 | 0 | 0.24 | 1 |
| 63 | KEGG_PATHOGENIC_ESCHERICHIA_COLI_INFECTION | 53 | 0.38 | 1.28 | 0 | 0.245 | 1 |
| 64 | KEGG_PROPANOATE_METABOLISM | 31 | -0.62 | -1.63 | 0 | 0.245 | 0.341 |
| 65 | KEGG_SPLICEOSOME | 126 | -0.44 | -1.61 | 0.088 | 0.197 | 0.341 |
| 66 | KEGG_ANTIGEN_PROCESSING_AND_PRESENTATION | 62 | -0.5 | -1.57 | 0 | 0.203 | 0.385 |
| 67 | KEGG_GRAFT_VERSUS_HOST_DISEASE | 31 | -0.46 | -1.57 | 0 | 0.177 | 0.433 |
| 68 | KEGG_VALINE_LEUCINE_AND_ISOLEUCINE_DEGRADATION | 42 | -0.54 | -1.56 | 0 | 0.167 | 0.433 |
| 69 | KEGG_RNA_DEGRADATION | 57 | -0.44 | -1.54 | 0 | 0.17 | 0.501 |
| 70 | KEGG_DNA_REPLICATION | 36 | -0.48 | -1.52 | 0 | 0.215 | 0.608 |
| 71 | KEGG_RNA_POLYMERASE | 28 | -0.5 | -1.52 | 0 | 0.203 | 0.662 |
| 72 | KEGG_BUTANOATE_METABOLISM | 30 | -0.42 | -1.52 | 0 | 0.188 | 0.662 |
| 73 | KEGG_CITRATE_CYCLE_TCA_CYCLE | 29 | -0.5 | -1.51 | 0.088 | 0.181 | 0.72 |
| 74 | KEGG_CYSTEINE_AND_METHIONINE_METABOLISM | 32 | -0.5 | -1.5 | 0 | 0.186 | 0.766 |
| 75 | KEGG_ONE_CARBON_POOL_BY_FOLATE | 17 | -0.55 | -1.5 | 0 | 0.183 | 0.828 |
| 76 | KEGG_CELL_CYCLE | 120 | -0.34 | -1.49 | 0 | 0.176 | 0.828 |
| 77 | KEGG_TYPE_I_DIABETES_MELLITUS | 37 | -0.42 | -1.49 | 0 | 0.17 | 0.828 |
| 78 | KEGG_ASCORBATE_AND_ALDARATE_METABOLISM | 15 | -0.58 | -1.48 | 0 | 0.173 | 0.828 |
| 79 | KEGG_FATTY_ACID_METABOLISM | 39 | -0.41 | -1.46 | 0 | 0.189 | 0.871 |
| 80 | KEGG_PEROXISOME | 73 | -0.32 | -1.45 | 0 | 0.183 | 0.871 |
| 81 | KEGG_STEROID_BIOSYNTHESIS | 17 | -0.54 | -1.45 | 0.137 | 0.176 | 0.871 |
| 82 | KEGG_DRUG_METABOLISM_OTHER_ENZYMES | 36 | -0.37 | -1.43 | 0.126 | 0.197 | 0.962 |
| 83 | KEGG_PARKINSONS_DISEASE | 116 | -0.48 | -1.43 | 0.131 | 0.19 | 0.962 |
| 84 | KEGG_VIRAL_MYOCARDITIS | 63 | -0.43 | -1.43 | 0 | 0.185 | 0.962 |
| 85 | KEGG_PYRUVATE_METABOLISM | 37 | -0.42 | -1.42 | 0 | 0.188 | 0.962 |
| 86 | KEGG_OOCYTE_MEIOSIS | 101 | -0.27 | -1.39 | 0 | 0.203 | 0.962 |
| 87 | KEGG_GLYCOLYSIS_GLUCONEOGENESIS | 52 | -0.34 | -1.37 | 0 | 0.231 | 1 |

**Table S4. Antibodies and commercial reagents used in this study.**

| **Antibodies/Regents** | **Source** | **Catalog Number** |
| --- | --- | --- |
| STAT5 antibody | CST | 94205T |
| STAT3 antibody | CST | 9139S |
| p-STAT5 (Tyr694) antibody | CST | 9359S |
| p-STAT3 (Tyr705) antibody | CST | 9145 |
| p65 antibody | Abclonal | A19653 |
| p-p65(Ser536) antibody | CST | 3033S |
| GAPDH antibody | Abcam | ab9385 |
| p53 antibody | Proteintech | 10442-1-AP |
| p-p53 (Ser15) antibody | CST | 9284S |
| P21 antibody | Proteintech | 10355-1-Ap |
| phospho-Histone H2A.X (Ser139) antibody | CST | 9718T |
| Lamin B1 antibody | Proteintech | #12987-1-AP |
| APC- anti-mouse- TER119 | eBioscience | 47-5921-80 |
| PE - anti-mouse- CD11b | Biolegend | 101207 |
| PB- anti-mouse- Ly-6G/Ly-6C | Biolegend | 108430 |
| PE - anti-mouse- CD41 | Biolegend | 133906 |
| PB- anti-mouse- Lineage | Biolegend | 133306 |
| Dual-Luciferase® Reporter Assay System | Promega | REF.E1910 |
| PrimerScript RT reagent | Takara | RR047A |
| SYBR Premix Ex Taq | Takara | RR420A |
| Cell-Light EDU Apollo567 In vitro Kit | RIBBIO | C10310-1 |

**Table S5. Primer sequences used in this study.**

| **Gene** | **Oligonucleotides** |
| --- | --- |
| Human-18S Primer | \| Forward: 5'-GCAATTATTCCCCATGAACG-3' \| \| --- \| \| Reverse: 5'-GGCCTCACTAAACCATCCAA-3' \| |
| Human-BCL2 Primer | \| Forward: 5'-ATCTGGGCCACAAGTGAAGT-3' \| \| --- \| \| Reverse: 5'-CAGCCTGCAGCTTTGTTTCAT-3' \| |
| Human-CCL2 Primer | \| Forward: 5'-CATGAAAGTCTCTGCCGCCC-3' \| \| --- \| \| Reverse: 5'-GGGCATTGATTGCATCTGGCTG-3' \| |
| Human-CCL5 Primer | \| Forward: 5'-CGTGCCCACATCAAGGAGTA-3' \| \| --- \| \| Reverse: 5'-TCGGGTGACAAAGACGACTG-3' \| |
| Human-CXCL10 Primer | \| Forward: 5'-CCACGTGTTGAGATCATTGCT-3' \| \| --- \| \| Reverse: 5'-TGCATCGATTTTGCTCCCCT-3' \| |
| Human-cyclinD1 Primer | \| Forward: 5'-GACCCCGCACGATTTCATTG-3' \| \| --- \| \| Reverse: 5'-AAATGAACTTCACATCTGTGGCA-3' \| |
| Human-HGF Primer  Human-IL-15 Primer | \| Forward: 5'-GACCCCGCACGATTTCATTG-3' \| \| \| --- \| --- \| \| Reverse: 5'-AAATGAACTTCACATCTGTGGCA-3' \| \| \| Forward: 5'-TCCATCCAGTGCTACTTGTGT-3' \| \| Reverse: 5'-CTGCACTGAAACAGCCCAAAA-3' \| |
| Human-IL-16 Primer | \| Forward: 5'-ATGATGCCTTGGCCATCCTC-3' \| \| --- \| \| Reverse: 5'-AGACTGTGGCCTCTGTAGATTC-3' \| |
| Human-IL-18 Primer | \| Forward: 5'-ATTGACCAAGGAAATCGGCCT-3' \| \| --- \| \| Reverse: 5'-TCCGGGGTGCATTATCTCTAC-3' \| |
| Human-IL-1βPrimer | \| Forward: 5'-AGCCATGGCAGAAGTACCTG-3' \| \| --- \| \| Reverse: 5'-CCTGGAAGGAGCACTTCATCT-3' \| |
| Human-Ki67 Primer  Human-MMP9 Primer  Human-p21 Primer  Human-PIM1 Primer  Human-TGF-β Primer  Human-TNF Primer | \| Forward: 5'-CTTTGGGTGCGACTTGACGA-3' \| \| --- \| \| Reverse:5'-ACAACTCTTCCACTGGGACG-3' \| \| Forward: 5'-CTTTGAGTCCGGTGGACGAT-3' \| \| Reverse:5'-TCGCCAGTACTTCCCATCCT-3' \| \| Forward: 5'-GCCGAAGTCAGTTCCTTGTG-3' \| \| Reverse:5'-TTCTGACATGGCGCCTCCT-3' \| \| Forward: 5'-TTATCGACCTCAATCGCGGC-3' \| \| Reverse:5'-TATACACTCGGGTCCCATCG-3' \| \| Forward: 5'-CAATTCCTGGCGATACCTCAG-3' \| \| Reverse:5'-GCACAACTCCGGTGACATCAA-3' \| \| Forward: 5'-CTGCACTTTGGAGTGATCGG-3' \| \| Reverse:5'-CTCAGCTTGAGGGTTTGCTACA-3' \| |
